# Selectively down-regulated PD-L1 by albumin-phenformin nanoparticles mediated mitochondrial dysfunction to stimulate tumor-specific immunological response for enhanced mild-temperature photothermal efficacy

**DOI:** 10.1186/s12951-021-01124-8

**Published:** 2021-11-18

**Authors:** Zaigang Zhou, Ning Jiang, Jiashe Chen, Chunjuan Zheng, Yuanyuan Guo, Ruirong Ye, Ruogu Qi, Jianliang Shen

**Affiliations:** 1 State Key Laboratory of Ophthalmology, Optometry and Vision Science, School of Ophthalmology and Optometry, School of Biomedical Engineering, Wenzhou Medical University, Wenzhou, 325027 China; 2 Department of Biochemistry and Molecular Biology, School of Medicine & Holistic Integrative Medicine, Nanjing University of Chinese Medicine, Nanjing, 210023 China; 3 Wenzhou Institute, University of Chinese Academy of Sciences, Wenzhou, 325001 China; 4 Faculty of Life Science and Technology, Kunming University of Science and Technology, Kunming, 650500 China; 5 Oujiang Laboratory (Zhejiang Lab for Regenerative Medicine, Vision and Brain Health), Wenzhou, 325001 Zhejiang China

**Keywords:** Mitochondrial inhibition, Mild-temperature photothermal therapy, Programmed cell death-ligand 1, Biomineralization, Tumor metastasis

## Abstract

**Background:**

Mild-temperature photothermal therapy (mild-PTT) has emerged as a highly promising antitumor strategy by triggering immunogenic cell death (ICD) to elicit both innate and adaptive immune responses for tumor control. However, mild-PTT still leads to the risk of tumor recurrence or metastasis because it could hardly completely eradicate tumors due to its impaired immunological efficacy owing to the enhanced PD-L1 expression in tumor cells after treatment.

**Results:**

In this study, we described a hydrogen peroxide (H_2_O_2_) responsive manganese dioxide mineralized albumin nanocomposite loading with mitochondria function inhibitor phenformin (PM) and near-infrared photothermal dye indocyanine green (ICG) by modified two-step biomineralization method. In combination with ICG induced mild-PTT and PM mediated mitochondria dysfunction, PD-L1 expression was obviously down-regulated and the generated immunological responses was able to effectively attack the remaining tumor cells. Meanwhile, the risk of tumor metastasis was effectively inhibited by reducing the expression of tumor invasion-related signal molecules (TGF-β and vimentin) after combining treatment.

**Conclusion:**

Such a strategy offers novel insight into the development of nanomedicine for mild-PTT as well as cancer immunotherapy, which can provide protection against tumor relapse post elimination of their initial and metastatic tumors.

**Graphical Abstract:**

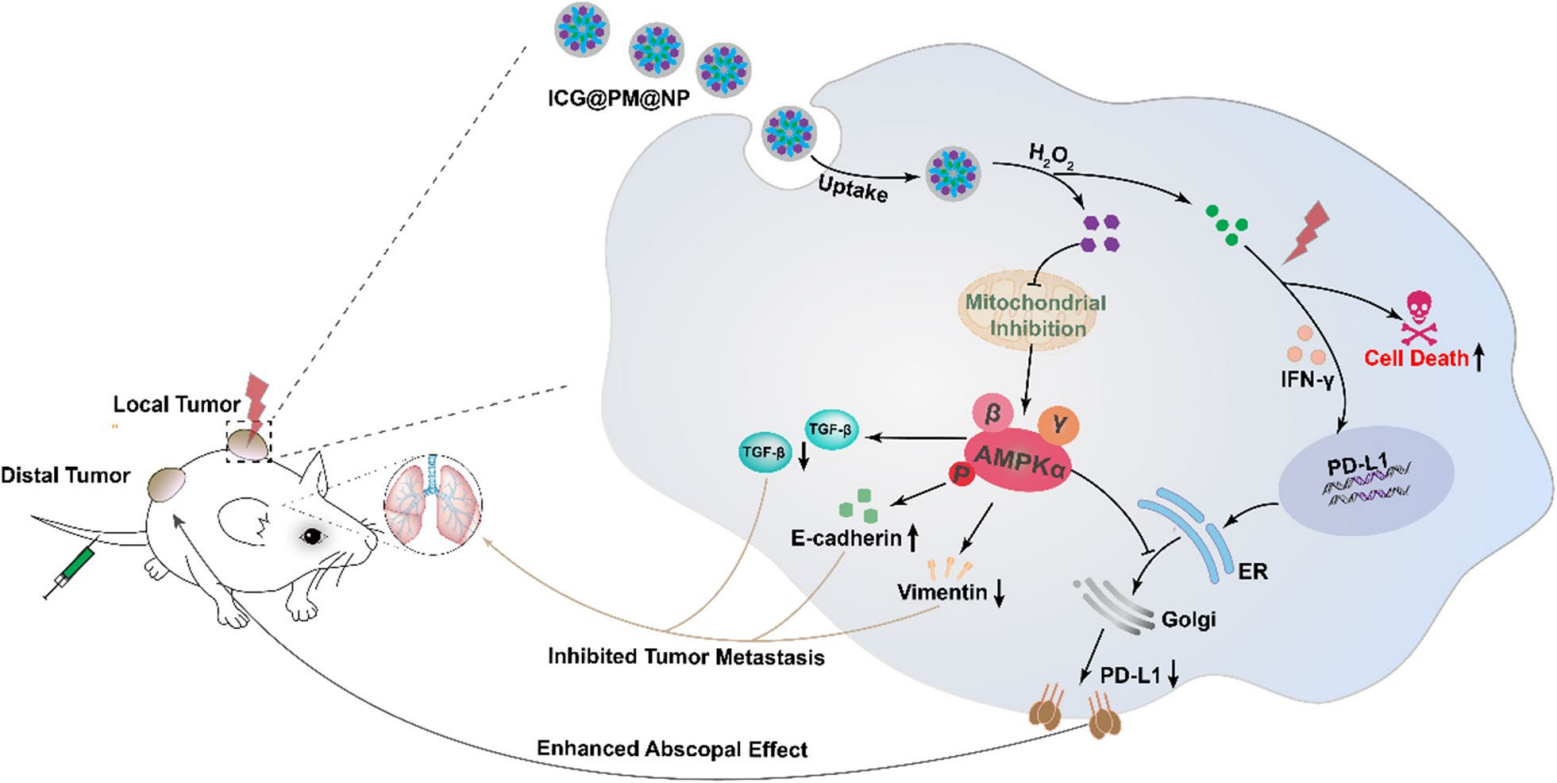

**Supplementary Information:**

The online version contains supplementary material available at 10.1186/s12951-021-01124-8.

## Highlights

- Over-expression of PD-L1 after mild-photothermal therapy significantly limited its efficacy.
- Phenformin could effectively downregulate PD-L1 expression and inhibit tumor metastasis through AMPK activation.
- Hydrogen peroxide responsive manganese dioxide mineralized albumin nanocomplex co-loading with phenformin and ICG named ICG@PM@NP was constructed by modified two-step biomineralization method.
- ICG@PM@NP could enhance T cell infiltration and antitumor metastasis in vivo.
- ICG@PM@NP mediated mild-photothermal therapy could make up the defects of conventional mild-photothermal therapy in lacking the anti-metastasis ability and inducing enhanced PD-L1 expression.

**Supplementary Information:**

The online version contains supplementary material available at 10.1186/s12951-021-01124-8.

## Introduction

Currently, developing effective cancer therapeutic strategies to selectively eliminate tumors with low toxicities are still urgently needed [1–3]. Conventional clinical therapies including chemotherapy, surgery, and radiotherapy are still limited with some fatal defects, including enhanced tumor metastasis and some other severe adverse effects [4–9]. To address these challenges, cancer immunotherapy exhibited enormous promises has been evolved as the next generation of therapeutic strategy. Training or stimulating the innate immunological systems to against tumor cells, immunotherapy provides an alternative for cancer treatment with effectiveness in tumor inhibition [10–12]. However, most of the universal immune-therapeutic strategies such as checkpoint blockade therapy, cytokine therapy, and adoptive T-cell transfer continue to confront limitations such as extremely high-cost, large individual variations in therapeutic responses, as well as certain immunotoxicity [13–16]. Therefore, cancer immunotherapy strategy that is feasible to operate and owns high specificity and safety is still urgently needed.

Recently, photothermal therapy (PTT), which utilizes the thermal ablation from the absorbed optical energy to inhibit primary tumor growth, has become an attractive cancer therapeutic option with the expectation to obtain satisfactory therapeutic outcomes [17–20]. In addition, the hyperthermia generated by PTT can trigger host immunity inducing immunogenic cell death (ICD), revealing a promising candidate for adjuvant cancer immunotherapy [21, 22]. Compared to the alternative immunotherapy strategy mentioned above, photothermal therapy (PTT) inducing ICD immunological therapy exhibited obvious advantages of high selective localized treatment, noninvasiveness, and controllable cost [11, 23]. However, high local hyperthermia (over 55 °C) during PTT treatment would inevitably result in serious damage to the neighboring healthy tissues and amplify the potential of tumor metastasis [21, 24, 25]. Thus, it is of clinical significance to perform PTT at a mild local temperature (below 50 °C) with increased security.

To make up for the defects of conventional PTT, mild-PTT with a relatively low temperature (below 50 °C) was applied in tumor treatment and considered as a critical variable in producing a favorable ICD for immunological responses [21, 23, 26]. Nevertheless, processing PTT-induced ICD at a mild local temperature (mild-PTT, around 45 °C) with reducing photothermal energy, the outcomes of this treatment are dramatically impaired [23, 27, 28]. Besides, such a mild temperature can also upregulate PD-L1 expression of tumor cells for facilitating tumor immunosuppressive microenvironment [3, 21, 23]. Moreover, the process of tumor metastasis, was also hardly inhibited or slowed down by mild-PTT [23, 29]. All in all, as an alternative to conventional PTT, the efficacy of mild-PTT still needs to be increased by co-treatment with antimetastatic therapy or anti-PD-L1 therapy [30].

Currently, AMP-activated protein kinase (AMPK) phosphorylation induced by metformin-mediated mitochondrial dysfunction has already been proved to possess the ability to decrease PD-L1 expression of tumor cells [31–33]. Subsequently, enhanced tumor immunogenetic activity could more effectively eliminate cancer cells [32]. Moreover, it was also reported that activation of AMPK by metformin could also be used as a potential strategy for prevention of cancer metastasis through disruption of E-cadherin-mediated cell–cell adhesion by blocking the expression of tumor invasion-related signaling molecules, such as TGF-β and Vimentin [34, 35]. However, such function of metformin could only be realized at an exceptionally high dosage of 100 mg/kg or even higher, which is almost impossible to achieve in the clinical [31, 36]. Up to now, few efforts have researched the possibility of using PD-L1 inhibitors to enhance the efficacy of mild-PTT except Liping Huang *et. al*. by co-using PD-L1 antibodies, the therapeutic effect of mild-PTT on inhibiting both primary and distal tumors was enhanced [23, 37]. Considering the possibility of metformin or its analogs in inhibiting PD-L1 and tumor metastasis simultaneously [32, 38], it has inspired us to propose that the combination of mild-PTT and metformin or its analogs will be a potential strategy to overcome the drawbacks of mild-PTT induced immunotherapy, which may increase the immunogenicity of tumors and inhibit tumor metastasis.

**Scheme 1.**
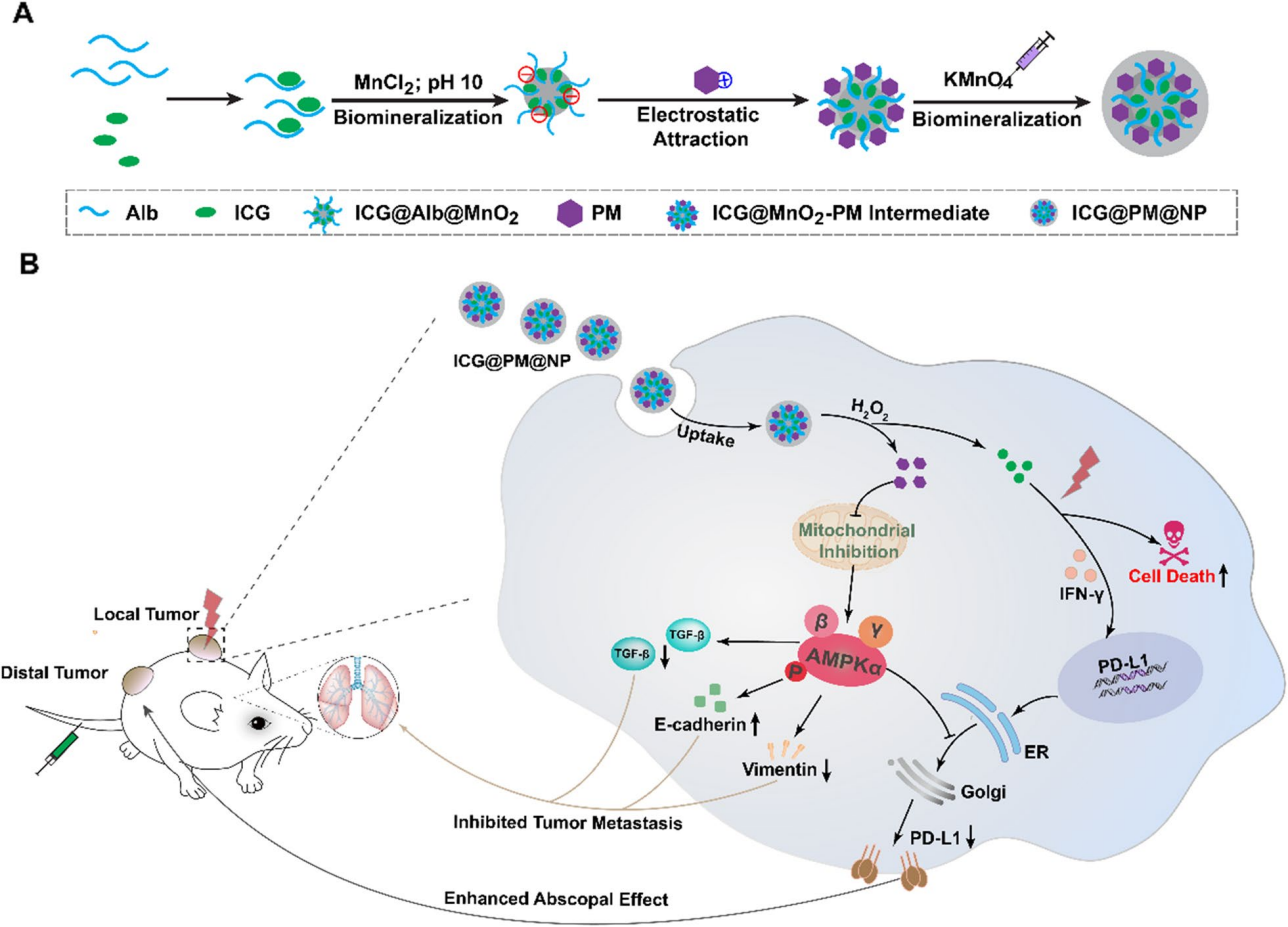
**A** The synthesis route of ICG@PM@NP nanoparticles by two-step bio-mineralization method. **B** The mechanism of enhanced mild-PTT efficacy induced by ICG@PM@NP mediated PD-L1 expression down-regulation and tumor metastasis inhibition through effective mitochondrial function inhibition

To validate the above hypothesis, hydrogen peroxide (H_2_O_2_) responsive manganese dioxide mineralized albumin (Alb) nanocomplex loading with photothermal agent ICG was prepared by modified two-step biomineralization method as the delivery platform for mild photothermal-sensitized immunotherapy (Scheme 1). Phenformin (PM), an analog of metformin, reported as a more effective AMPK activator to overwhelm the defects of metformin was also encapsulated to the nanocomplex as an adjuvant to enhance the immuno-response of tumor cells (Scheme 1A) [27, 28]. After tail vein injection of this nanocomplex, the release of ICG and PM could be triggered by the degradation of manganese dioxide due to the highly H_2_O_2_ concentration at the tumor microenvironment. The released ICG could then generate mild increase in the temperature for mild-PTT and activate the systemic immune response for ICD under manually controlled NIR irradiation. Meanwhile, PM would induce the AMPK phosphorylation via tumor mitochondrial function inhibition subsequently to down-regulate PD-L1 protein, maximized the tumor-specific immune responses of ICG mediated mild-PTT to against the abscopal tumors. Moreover, the risk of tumor metastasis could then be reduced by downregulating the expression of TGF-β and Vimentin due to the AMPK phosphorylation (Scheme 1B). In this way, these properties implied that the Alb-PM nanoplatform could work as a general strategic platform for the desired mild-PTT.

## Results

### PM down-regulated PD-L1 expression by inducing AMPK phosphorylation

**Fig. 1:**
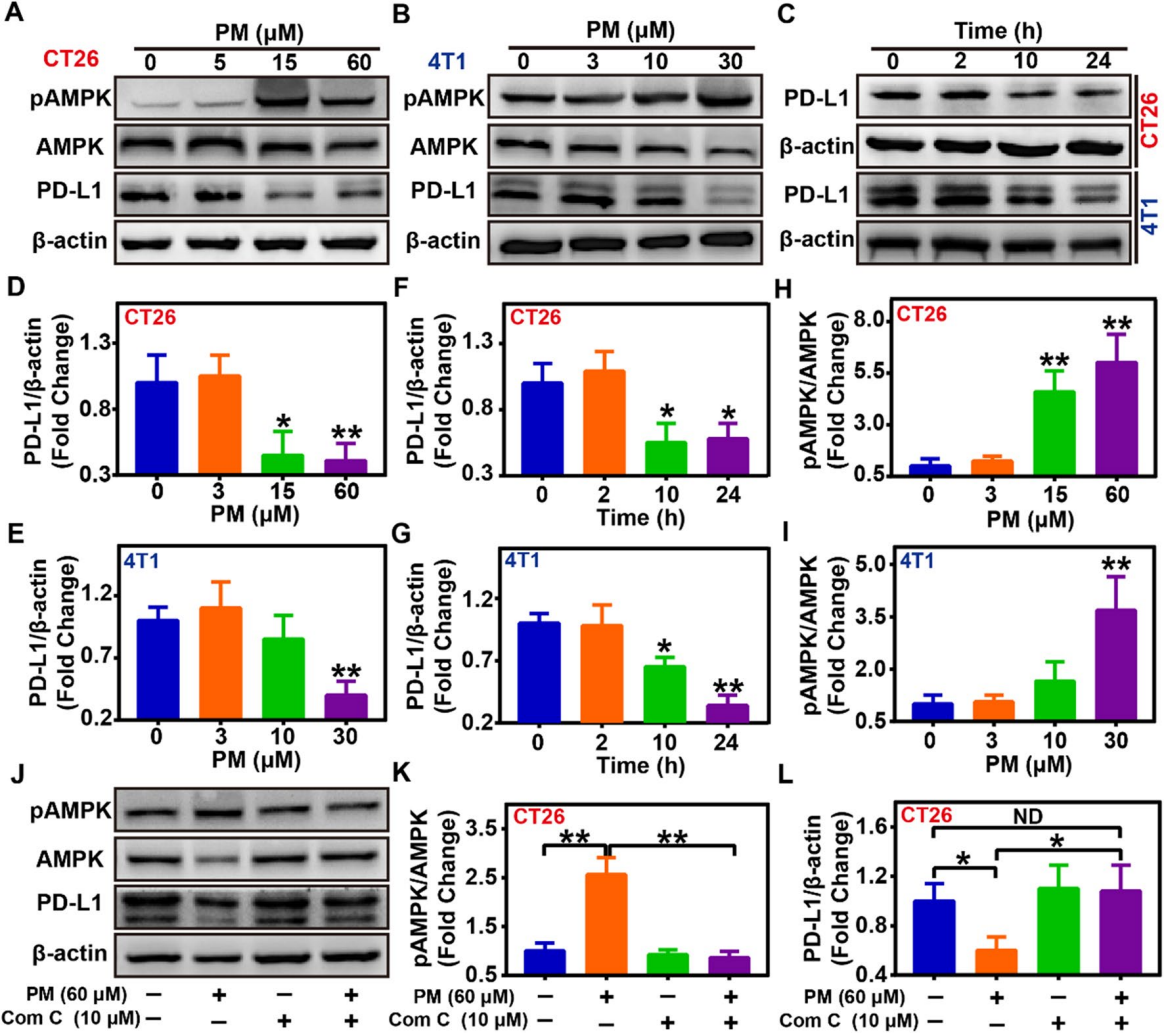
Effects and mechanism of PM on PD-L1 expression in different tumor cells*.* **A**, **B** Detection of PD-L1, pAMPK, and AMPK protein by western blot in CT26 and 4T1 cells after treatment by different concentrations of PM for 24 h (n = 3). **C** Detection of PD-L1 protein expression in CT26 and 4T1 cells by western blot after 30 μM PM treatment for different time (n = 3). **D**–**G** Quantification of PD-L1 protein in CT26 or 4T1 tumor cells in western blot assay by ImageJ (n = 3). **H**, **I** Quantification of pAMPK and AMPK protein in western blot assay by ImageJ in CT26 and 4T1 cells after treatment with different concentrations of PM for 24 h (n = 3). **J**–**L** Detection and quantification of PD-L1, pAMPK, and AMPK protein in CT26 cells by western blot after 60 μM PM treatment in the presence or absence of 10 μM AMPK phosphorylation inhibitor Compound C for 24 h (n = 3). Data were demonstrated as mean ± SD. Statistical analysis was performed via the two-tail Student’s *t*-test. ND, no significant difference; * *p* < 0.05; ** *p* < 0.01

AMPK activation induced by metformin-mediated mitochondrial function inhibition has been newly reported to phosphorylate Serine 195 of PD-L1, leading to the degradation of PD-L1 [31, 32]. PM, a metformin analog, used as an antidiabetic or anticancer drug, has been proved to possess more obvious AMPK activation ability than metformin at a far lower dose (30 μM *vs* 2 mM) [38, 39]. However, the capacity of PM in regulating PD-L1 expression for tumor cells as metformin still needs to be confirmed [38, 39]. Thus, the ability and mechanism of free PM in regulating PD-L1 expression were firstly evaluated. As results indicated, it was revealed that PM could dose-dependently induce AMPK phosphorylation (Fig. 1A, B, H, I). As reported, the activation of AMPK could then lead to the lower expression of the PD-L1 protein. On the one hand, dose-dependently down-regulated PD-L1 expression mediated by PM in CT26 and 4T1 tumor cells was observed, which was about 41.2 ± 11.2% of the basal PD-L1 expression level as 60 μM PM pretreatment in CT26 cells and 24.5 ± 14.7% as 30 μM PM pretreatment in 4T1 cells (Fig. 1A, B, D, E)*.* On the other hand, PM also could time-dependently decrease the PD-L1 expression in CT26 and 4T1 tumor cells (Fig. 1C, F, G)*.* To assess whether the decreased PD-L1 expression induced by PM was mediated by enhanced AMPK activation, the AMPK phosphorylation levels after PM treatment was evaluated, showing a dose-dependently increase (Fig. 1A, B, H, I). Meanwhile, cells were co-treated with PM and 10 μM AMPK inhibitor Compound C to hinder AMPK binding phosphorylation. Notably, Compound C effectively antagonized the effect of PM-related PD-L1 down-regulation (Fig. 1J–L). Finally, the effects of PM on the PD-L1 expression in MCF-7 human tumor cells in vitro was also evaluated, showing that PM could dose-dependently induce the phosphorylation of AMPK protein and reduce PD-L1 expression in human tumor cells (Additional file 1: Fig. S2). Collectively, these results above indicated that PM could induce the lower expression of PD-L1 protein through AMPK signaling activation with a far lower dose compared with its analog metformin both in mice and human cancer cells (Fig. 1; Additional file 1: Fig. S2).

### Preparation and characterization of ICG@PM-NPs

**Fig. 2:**
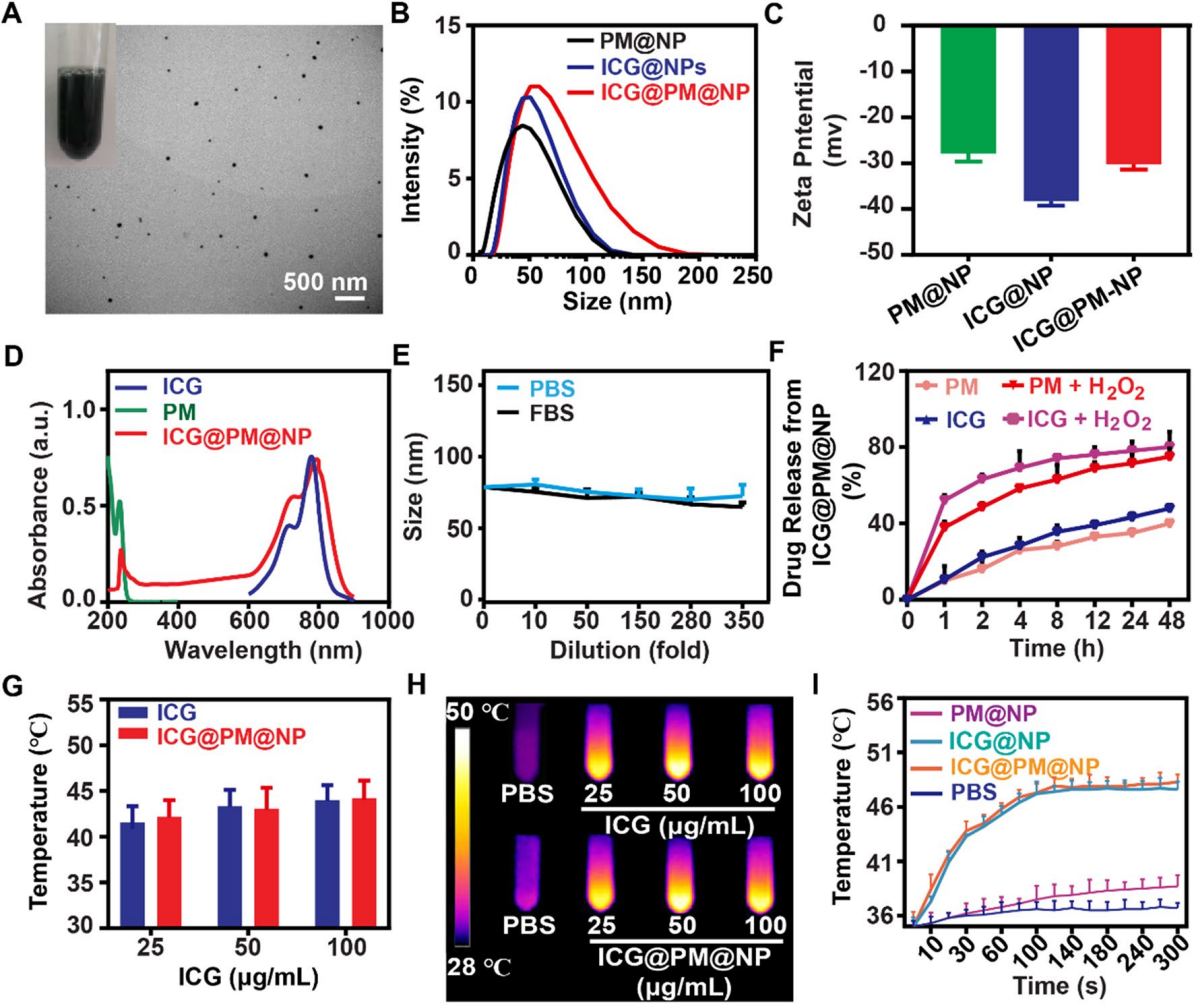
Characterization of the ICG@PM@NP. **A** Representative TEM image of the prepared ICG@PM@NP, scale bar = 500 nm. **B** Hydrodynamic diameters of ICG@NP, PM@NP, and ICG@PM@NP. **C** Zeta potential of PM@NP, ICG@NP, and ICG@PM@NP (n = 3). **D** UV–VIS spectra of free ICG, PM, and ICG@PM@NP in deionized water. **E** The stability of ICG@PM@NP in PBS and FBS was detected by DLS (n = 3). **F** Release of PM and ICG from ICG@PM@NP in PBS or PBS plus 100 μM H_2_O_2_ (n = 3). **G**–**H** Thermal images and temperature variation of different concentrations of free ICG or ICG@PM@NP (calculated by ICG content) exposed to 1 W/cm^2^ laser for 60 s (n = 3). **I** Temperature variation curves of the aqueous dispersion of PBS, ICG@NP, PM@NP, and ICG@PM@NP exposed to 1 W/cm^2^ laser for different time (n = 3). Data were demonstrated as mean ± SD. Statistical analysis was performed via the two-tail Student’s *t*-test

The ability of PM in downregulating PD-L1 expression has been confirmed as above. However, the serious toxicity of PM like acidosis still obviously limits its widespread usage [27, 38]. To effectively deliver ICG and PM in the tumors to obtain ideal drug accumulation and avoid possible side effects, ICG@PM@NP nanocomplexe was prepared by loading ICG and PM into Alb@MnO_2_ composite through the modified two-step bio-mineralization method (Scheme 1A) [24, 40–42]. Typically, MnCl_2_ was reduced by the ICG-Alb complex to obtain ICG@Alb@MnO_2_, in which PM could bind with ICG@Alb@MnO_2_ through electrostatic absorption. Then, KMnO_4_ was reduced by Alb to form MnO_2_ nano-shell on its surface for further stabilizing ICG@PM@NP to avoid possible drug leakage (Scheme 1A) [24, 43, 44]. The stable prepared ICG@PM@NP showed green-dark color and homogeneous spherical structure as observed by TEM (Fig. 2A). The hydrodynamic diameter and surface charge of nanocomplexes were further measured. As shown in Fig. 2B, C, compared to the IGG@NP, ICG@PM@NP exhibited a larger size (63.5 ± 4.7 nm *vs* 55.9 ± 6.4 nm) and higher zeta potential (− 30.5 ± 1.6 mV *vs* − 38.9 ± 2.1 mV) due to the encapsulation of cationic hydrophobic drug PM (Fig. 2A–C). The successful loading of PM was confirmed through UV–VIS spectrometry by the overlap of peaks among ICG@PM@NP, small molecule PM and ICG (Fig. 2D). The loading capacity of ICG and PM in ICG@PM@NP were calculated through UV–VIS absorbance as 75.2 ± 8.9% and 45.6 ± 7.4%, respectively. Generally, the stability of nanocomplexes was vital for its in vivo delivery efficacy. The stability of ICG@PM@NP was further measured by the dilution method. As Additional file 1: Fig. S3 showed, the particle size of ICG@PM@NP was not changed in both PBS buffer, 10% FBS and water at different time. Besides, the particle size of ICG@PM@NP remained unchanged even diluted for 350-fold, showing favorable stability (Fig. 2E). Following this, the responsive release behavior of ICG@PM@NP was also tested. ICG and PM exhibited a burst releasing profile of PM and ICG (around 50% payload releasing in 1 h) in the presence of 100 μM H_2_O_2_ PBS buffer compared to that of in blank PBS (less than 15% payload releasing in 1 h) (Fig. 2F). Results also showed that the release of ICG from ICG@PM@NP in PBS buffer at pH 5.5 rather than PBS buffer at pH 6.5 was obviously enhanced compared with PBS buffer at pH 7.6, meaning that the acidic environment of tumor lysosomes may also promote the release of ICG from ICG@PM@NP (Additional file 1: Fig. S4). All these results revealed that ICG@PM@NP possessed ideal particle size distribution, favorable particle stability, and desired H_2_O_2_ responsive drug releasing behavior.

Subsequently, the mild photothermal effects of ICG@PM@NP were evaluated. After irradiation for 60 s at 1 W/cm^2^ with 808 nm laser, ICG@PM@NP solution at 25 μg/mL to 100 μg/mL can generate a temperature increase from 30 to 42 ℃, respectively (Fig. 2G, H). Then, the hyperthermia efficacy of ICG@PM@NP solution (25 μg/mL) with 808 nm laser at various time points was also studied. As Fig. 2I shown, no obvious irritation hyperthermia effect was observed in PM@NP while ICG@PM@NP reached the temperature of nearly 45 ℃ for 60 s irradiation, same as ICG @NP. After multiple irradiations, the rising extent of temperature gradually declined after every irradiation, since irradiation induced the degradation of ICG (Additional file 1: Fig. S5). All in all, these results verified that ICG@PM@NP was capable of generating stable mild hyperthermia.

### ICG@PM@NP improved the efficacy of mild-PTT and decreased PD-L1 expression in vitro

**Fig. 3:**
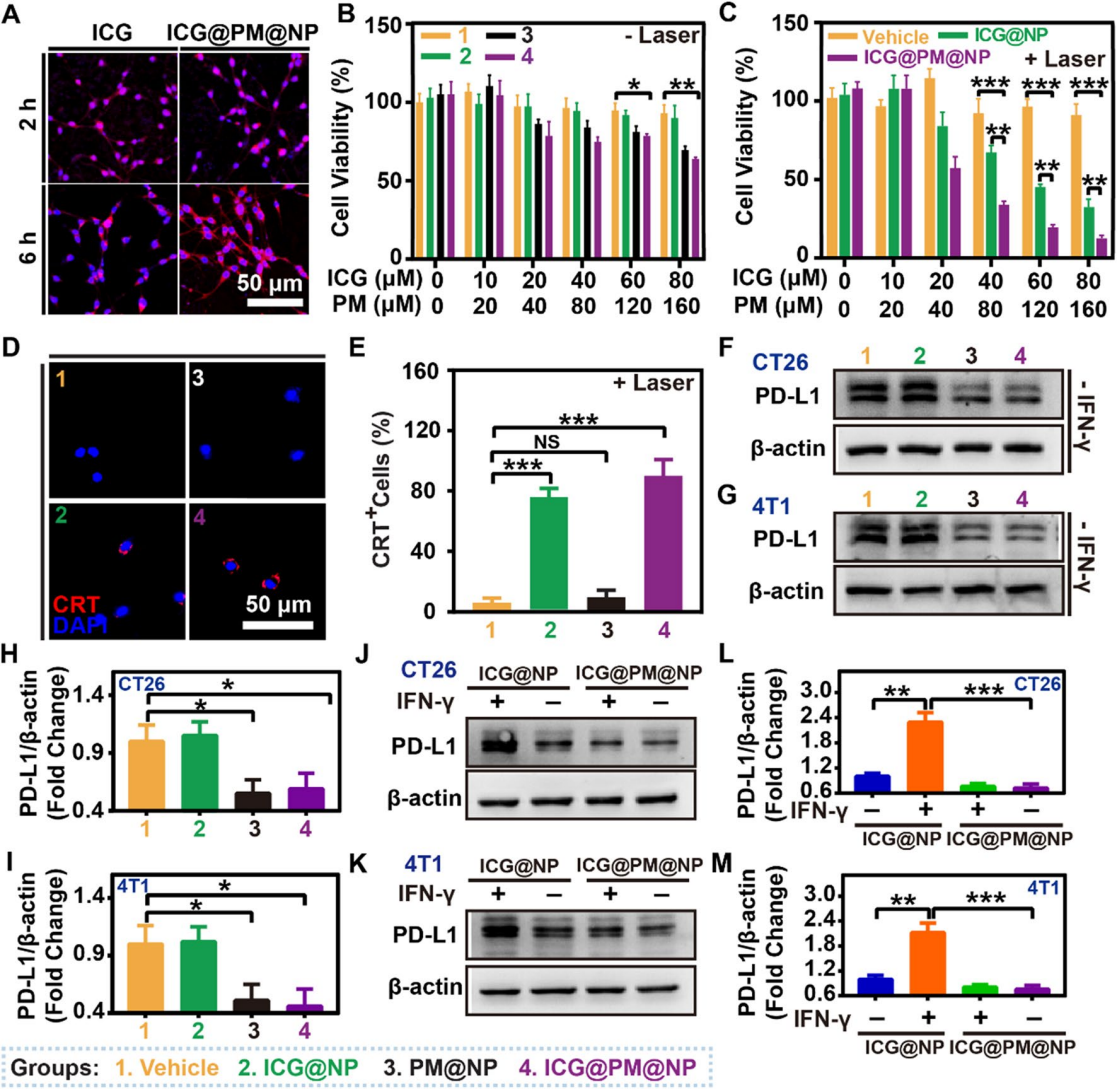
ICG@PM@NP improved the efficacy of mild-PTT and decreased PD-L1 expression in vitro. **A** Representative fluorescence image of ICG@PM@NP accumulation in CT26 cells, scale bar = 50 μm. **B**, **C** Cell viability of CT26 cells detected by CCK-8 assay (n = 3). **D** Representative immunofluorescence images of ICG@PM@NP mediated CRT exposure after mild-PTT treatment, scale bar = 50 µm. **E** Quantification of ICG@PM@NP mediated CRT exposure after mild-PTT treatment (n = 3). **F**–**G** Effects of ICG@PM@NP on the basal PD-L1 protein expression in CT26 and 4T1 cells detected by western blot assay after treatments for 24 h (n = 3). **H**, **I** Quantification of PD-L1 protein expression levels in western blot assay by ImageJ in CT26 and 4T1 cells (n = 3). **J**, **K** Effects of ICG@PM@NP on the IFN-γ induced over-expression of PD-L1 protein in CT26 and 4T1 cells after treatments for 24 h (n = 3). **L**, **M** Quantification of the PD-L1 protein expression levels by ImageJ in CT26 and 4T1 cells (n = 3). Data were demonstrated as mean ± SD. Statistical analysis was performed via the two-tail Student’s *t*-test. ND, no significant difference; * *p* < 0.05; ** *p* < 0.01; *** *p* < 0.001

To determine the hyperthermia effect of ICG@PM@NP in vitro, the cell uptake of free ICG and ICG@PM@NP in CT26 tumor cells was evaluated. As shown in Fig. 3A and Additional file 1: Fig. S6, a similar intensity of ICG fluorescence signal was observed inside the CT26 cell cytoplasm after incubation of free ICG or ICG@PM@NP for 2 h and 6 h, indicating that ICG@PM@NP showed similar cell uptake as free ICG. Then, the hyperthermia efficacy of ICG@PM@NP to cause CT26 cell death was tested in vitro in the presence or absence of NIR. As Fig. 3B shown, various concentrations of ICG@NP itself exhibited little cytotoxicity without NIR. With the increase of PM concentration, the cytotoxicity of PM@NP or ICG@PM@NP treated cells was slightly enhanced (Fig. 3B). Notably, when ICG@NP was treated with NIR, mild-PTT mediated by ICG@NP showed more obvious cytotoxicity with the increase of ICG concentration than ICG@NP treated alone (Fig. 3C). Unlike the mild-PTT mediated by ICG@NP alone, the increase in PM concentration after the co-treatment of ICG@PM@NP and NIR caused the more obvious cytotoxicity (Fig. 3C). Thus, ICG@PM@NP mediated mild-PTT had the most obvious cytotoxicity. When tumor cells experience PTT-induced immunogenic death, the expression of signal molecules on the cell membrane surface, including the release of over-expressed calreticulin (CRT) could be observed. As shown in Fig. 3D–E, CRT valgus to the tumor cell membrane was also significantly enhanced after ICG@PM@NP or ICG@NP mediated mid-PTT treatment, indicating that the immunogenic death of tumor cells was also amplified. In summary, these results consistently proved that mild-PTT mediated by ICG@PM@NP had a more pronounced antitumor effect than mild-PTT mediated by ICG@NP alone.

As demonstrated above, AMPK activation mediated by PM could lead to decreased PD-L1 expression in tumor cells (Fig. 1), indicating that PM@NP or ICG@PM@NP loading with PM may also possess the ability to reduce PD-L1 expression. After treating with ICG@PM@NP for CT26 and 4T1 cells, the expression of PD-L1 in tumor cells was reduced to about 50% of the basal expression level, while ICG@NP itself did not have this ability (Fig. 3F–I). Similar to the results indicated in mice tumor cells, ICG@PM@NP also possessed the ability to decrease the PD-L1 expression in human tumor cells (Additional file 1: Fig. S7). Then, IFN-γ, the most effective inducer of PD-L1 expression secreted by activated T cells in ICD cascade, was also selected to evaluate the possible ability of ICG@PM@NP to reverse the PD-L1 upregulation induced by activated T cells. As Fig. 3J–M showed, the PD-L1 expression levels after IFN-γ and ICG@NP co-treatment was increased about 2.2-fold compared with ICG@NP treated alone in both CT26 cells and 4T1 cells, which was then inhibited by co-treating IFN-γ with ICG@PM@NP or ICG@PM@NP alone. Recently, it has been newly discovered that mild-PTT can increase the PD-L1 expression to a certain extent. [12, 16] Therefore, mild-PTT mediated by ICG@PM@NP may also compensate for the defect of ICG@NP mediated mild-PTT in reversing the overexpression of PD-L1. Further, we confirmed that the PD-L1 expression level after treating with laser irritating ICG@NP induced mild-PTT was approximately 1.4 times higher than that of ICG@NP treated alone (Additional file 1: Fig. S8). In contrast, for mild-PTT mediated by ICG@PM@NP, this phenomenon was effectively reversed due to the ability of PM in inhibiting PD-L1 expression (Additional file 1: Fig. S8). These results fully proved that mild-PTT mediated by ICG@PM@NP can compromise the disadvantages of conventional mild-PTT in leading to the overexpression of PD-L1 protein.

### ICG@PM@NP effectively inhibited tumor cell metastasis

**Fig. 4:**
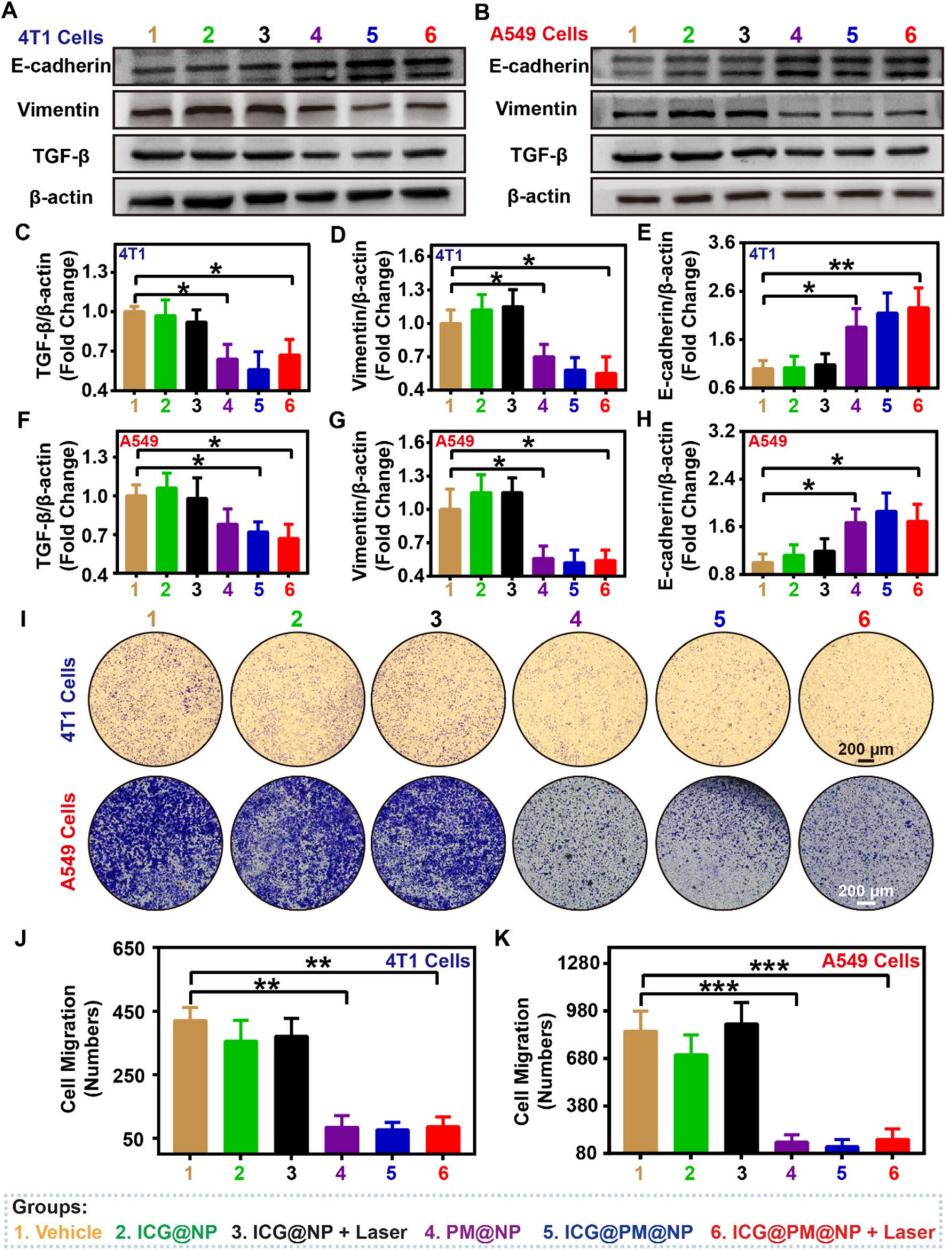
ICG@PM@NP effectively inhibited tumor cell migration and metastasis. **A**, **B** Effects of ICG@PM@NP on the TGF-β, E-cadherin, and Vimentin protein expression in 4T1 and A549 cells detected by western blot assay after different treatments for 24 h (n = 3). **C**–**H** Quantification of TGF-β, E-cadherin, and Vimentin protein expression levels in western blot by ImageJ in 4T1 cells and A549 cells (n = 3). **I**–**K** Representative images and quantitative analysis of the migration of 4T1 cells and A549 cells after different treatments for 24 h (n = 3), scale bar = 200 µm. Data were demonstrated as mean ± SD. Statistical analysis was performed via the two-tail Student’s *t*-test. * *p* < 0.05; ** *p* < 0.01; *** *p* < 0.001

Recently, studies have shown that upregulation of pAMPK can inhibit cancer cell migration and epithelial-mesenchymal transition (EMT) by regulating the classical transforming growth factor β (TGF-β) signaling pathway, resulting in the increased expression of E-cadherin and decreased expression of vimentin [20]. Considering the ability of PM in inducing the up-regulation of pAMPK (Fig. 1), ICG@PM@NP may also could inhibit tumor metastasis. To prove this, the expression levels of TGF-β, E-cadherin, and vimentin after ICG@PM@NP treatment were elevated by western blot analysis (Fig. 4). As Fig. 4A–H shown, PM@NP and ICG@PM@NP could reduce the expression of TGF-β and vimentin in 4T1 mouse cells and A549 human cells to about 60% of that in the vehicle group. Besides, the expression of another tumor metastasis marker E-cadherin was also increased to 1.7–2.0 fold that of the vehicle group. In addition, the expression of these tumor metastasis protein markers exhibited no obvious regulation by ICG@NP alone or mild-PTT mediated by ICG@NP (Fig. 4A–H). To further evaluate the potential impact of ICG@PM@NP or mild-PTT mediated by ICG@PM@NP on cancer metastasis, the Boyden chamber invasion test using 4T1 breast cancer cell line and A549 lung cancer cell line was conducted (Fig. 4I–K). As Fig. 4I–K showed, PM@NP, ICG@PM@NP, or ICG@PM@NP mediated mild-PTT (PM concentration: 30 μM) significantly inhibited cell migration, while ICG@NP mediated mild-PTT did not have this effect. Importantly, the anti-migration effect of ICG@PM@NP mediated mild-PTT was observed at a concentration lower than the anti-proliferative effect (PM concentration: 30 μM *vs* more than 40 μM) (Fig. 4I–K). Therefore, the anti-migration effect of mild-PTT mediated by ICG@PM@NP could effectively inhibit tumor metastasis, not due to the inhibited cell proliferation mediated by PM.

### Pharmacokinetics and bio-distribution of ICG@PM@NP

**Fig. 5:**
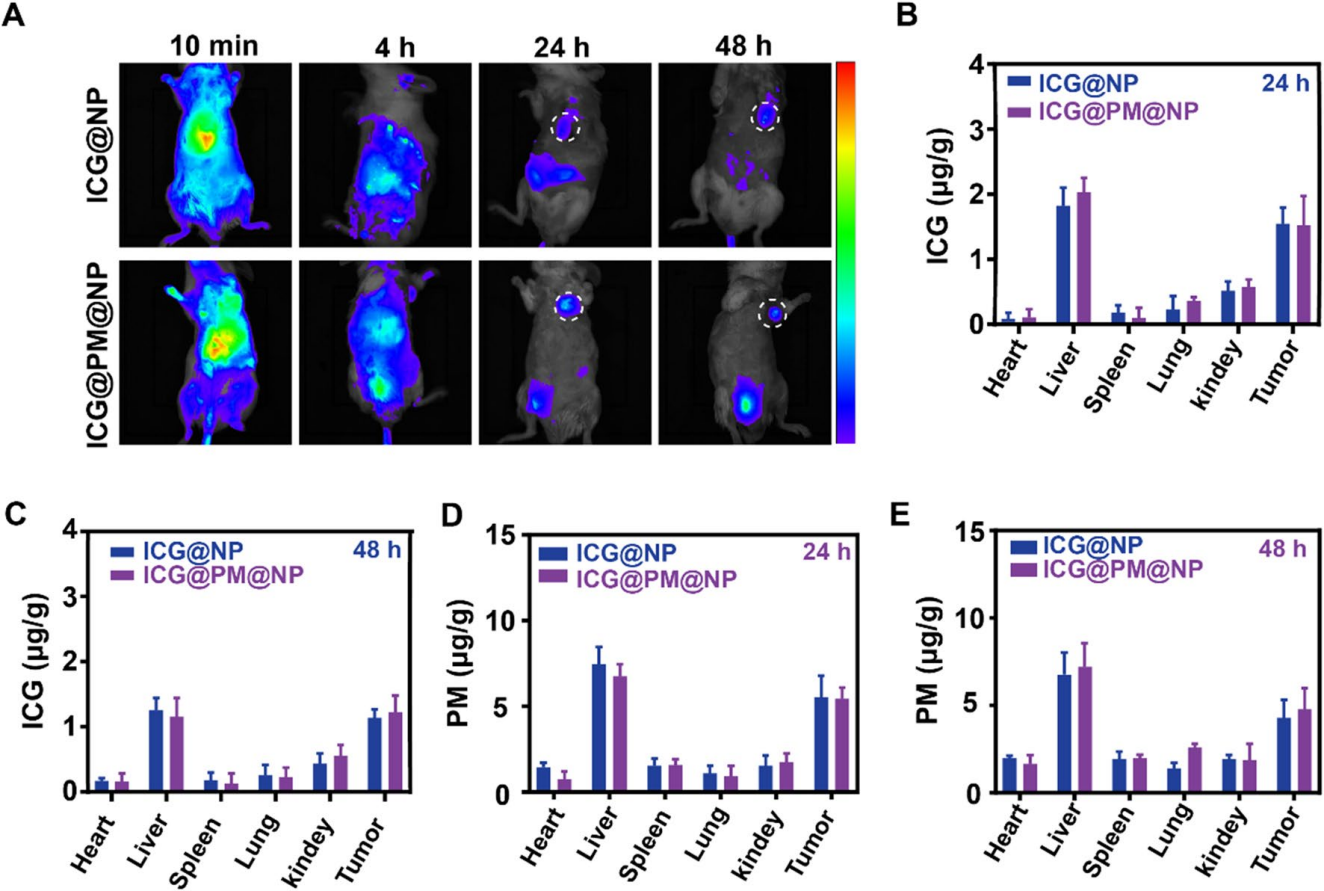
Pharmacokinetics and bio-distribution of ICG@PM@NP in vivo. **A** Real-time NIR fluorescence images of the CT26 tumor-bearing mice after intravenous injection of ICG@NP or ICG@PM@NP at different time points. **B**–**E** Quantification of ICG or PM concentration in tumors and normal tissues at 24 h or 48 h after ICG@NP or ICG@PM@NP treatments (n = 3). Data were demonstrated as mean ± SD. Statistical analysis was performed via the two-tail Student’s *t*-test

To investigate the pharmacokinetics of ICG@PM@NP, in vivo real-time near-infrared fluorescence imaging analysis of ICG@PM@NP dosing mice was performed. After ICG@PM@NP or ICG@NP administration, the distribution of ICG in CT26 tumors increased significantly over time (Fig. 5A). At 24 and 48 h, more ICG fluorescence signal was observed in the tumors of ICG@PM@NP or ICG@NP treated mice when compared with other normal tissues like heart and lung, which may be induced by enhanced permeability and retention effect and the active tumor-targeting ability of albumin (Fig. 5A).[24, 40, 45] Following this, UV–VIS and HPLC–MS were used to more accurately evaluate the accumulation of ICG and PM in tumors, respectively (Fig. 5B–E). As Fig. 5B, C indicated, compared with normal tissues such as spleen, heart, lung, and kidney, about 4–fivefold ICG were accumulated in the tumors at 24 h or 48 h after ICG@PM@NP or ICG@NP treatment. Meanwhile, the accumulation of PM in tumors was also about 2.5–fivefold higher than its accumulation in the spleen, heart, lung, and kidney at 24 h or 48 h after ICG@PM@NP or ICG@NP treatment (Fig. 5D, E). Therefore, when encapsulated in ICG@PM@NP, ICG can better accumulate at the tumor site instead of being quickly eliminated from mice like free ICG (Fig. 5B–E). In addition, the enhanced accumulation of PM in tumors rather than other tissues may better avoid the toxic side effects of free PM. Thus, ICG@PM@NP with ideal tumor accumulation is more suitable for the needs of high-efficiency, non-toxic mild-PTT.

### Effects of ICG@PM@NP nanoparticles on PD-L1 protein expression, T cell infiltration, and cell apoptosis in vivo

**Fig. 6:**
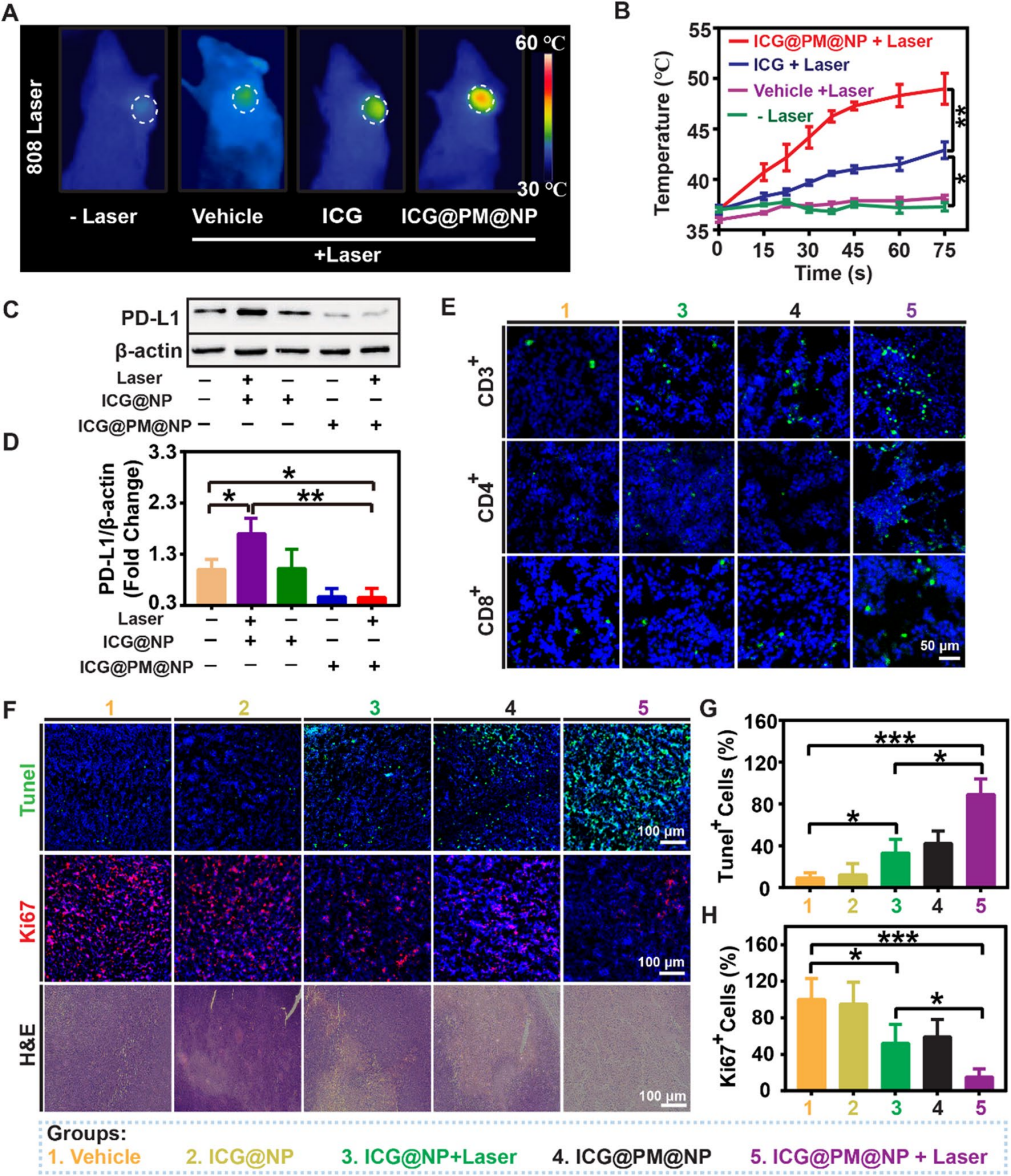
Effects of ICG@PM@NP on PD-L1 protein expression, T cell infiltration, and cell apoptosis in vivo. **A** Representative in vivo infrared thermal images of CT26 tumors irradiated 60 s at 1 W/cm^2^ for twice at 24 h after different treatments. **B** The temperature curves of CT26 tumors after NIR laser irradiation for different time, the mice were pre-treated with different drugs for 24 h (n = 3). **C**, **D** PD-L1 expression detection and quantification in CT26 tumors by western blot (n = 3). **E** Representative fluorescence images of CD3^+^ T cells, CD8^+^ T cells, and CD4^+^ T cells in CT26 tumor slices to evaluate the effects of ICG@PM@NP mediated mild-PTT on T cell infiltration, scale bar = 50 µm. **F** Representative images of H&E staining, Ki67 staining, and Tunel staining, scale bar = 100 µm. **G**–**H** Quantification of Tunel^+^ and Ki67^+^ tumor cells (n = 3). Data were demonstrated as mean ± SD. Statistical analysis was performed via the two-tail Student’s *t*-test. * *p* < 0.05; ** *p* < 0.01; *** *p* < 0.001

To evaluate the effect of mild-PTT mediated by ICG@PM@NP in CT26 tumor-bearing mice in vivo, full-body thermal imaging was used to measure the changes of temperature in CT26 tumors. The temperature of CT26 tumors increased significantly within 60 s after ICG@PM@NP including PTT (from 36.5 ± 0.4 ℃ to 47.3 ± 2.4 ℃) (Fig. 6A, B; Additional file 1: Fig. S9). In addition, mice treated with free ICG did not induce such a high tumor temperature increase (from 36.7 ± 0.3 ℃ to 41.5 ± 1.1 ℃) due to the limited ICG accumulation in the tumors as the form of free ICG (Fig. 6A, B). Therefore, ICG@PM@NP co-treated with NIR could induce ideal mild-PTT in vivo.

After mild-PTT, the expression of IFN-γ protein, the most effective PD-L1 expression inducer secreted by activated T cells, may also further increase PD-L1 expression in tumors. Additionally, the upregulated expression of PD-L1 after mild-PTT has been demonstrated in vitro (Fig. 3). These two aspects may synergistically lead to the overexpression of PD-L1 in tumors [34]. Therefore, we speculated that mild-PTT mediated by ICG@NP may also increase the expression of PD-L1 protein in vivo. As expected, the expression level of PD-L1 after mild-PTT mediated by ICG@NP was about 1.7 times that of the vehicle group (Fig. 6C, D). When treated with mild-PTT mediated by ICG@PM@NP, this phenomenon was reduced to about 50% of the PD-L1 expression level compared with the vehicle group (Fig. 6C, D). Furthermore, considering the proven fact that mild-PTT mediated by ICG@PM@NP can significantly inhibit the expression of PD-L1 in vivo, T cell infiltration in tumors may also be significantly enhanced. As speculated, compared with the vehicle group, the distribution of CD3^+^, CD4^+^ and CD8^+^ T cells in the tumor were slightly enhanced after ICG@PM@NP treatment (Fig. 6E; Additional file 1: Fig. S10). In addition, compared with the conventional mild-PTT like ICG@NP, T cell infiltration in CT26 tumors was also significantly enhanced after ICG@PM@NP mediated mild-PTT treatment (Fig. 6E; Additional file 1: Fig. S10). These results suggested that mild-PTT mediated by ICG@PM@NP reversed the tumor immunosuppressive microenvironment more effectively by regulating PD-L1 expression compared to the conventional mild-PTT.

Encouraged by the results above, mild-PTT mediated by ICG@PM@NP may lead to more obvious tumor cell death in vivo by enhancing immune killing induced by T cell infiltration and chemical killing caused by elevated tumor temperature. To prove this, Ki67, a proliferative cell-expressed protein that closely related to mitosis and TdT-mediated dUTP Nick-End Labeling (Tunel), a method to detect DNA breakage to reflect the tumor cell apoptosis, were applied to evaluate the efficacy of mild-PTT mediated by ICG@PM@NP in inducing tumor cell death (Fig. 6). As Fig. 6F–G shown, compared with mild-PTT mediated by ICG@NP, mild-PTT mediated by ICG@PM@NP killed more CT26 tumor cells (86.9 ± 15.7% vs 33.4 ± 13.8%). At the same time, when treated with mild-PTT mediated by ICG@PM@NP, the proliferation of the remaining tumor cells was almost completely inhibited, while mild-PTT mediated by ICG@NP not (15.6 ± 8.9% vs 52.6 ± 21.3%) (Fig. 6F, H). Therefore, mild-PTT mediated by ICG@PM@NP can most effectively inhibit tumor cell growth and cause tumor cell death in vivo.

### In vivo antitumor efficacy of ICG@PM@NP mediated mild-PTT in CT26 tumors

**Fig. 7:**
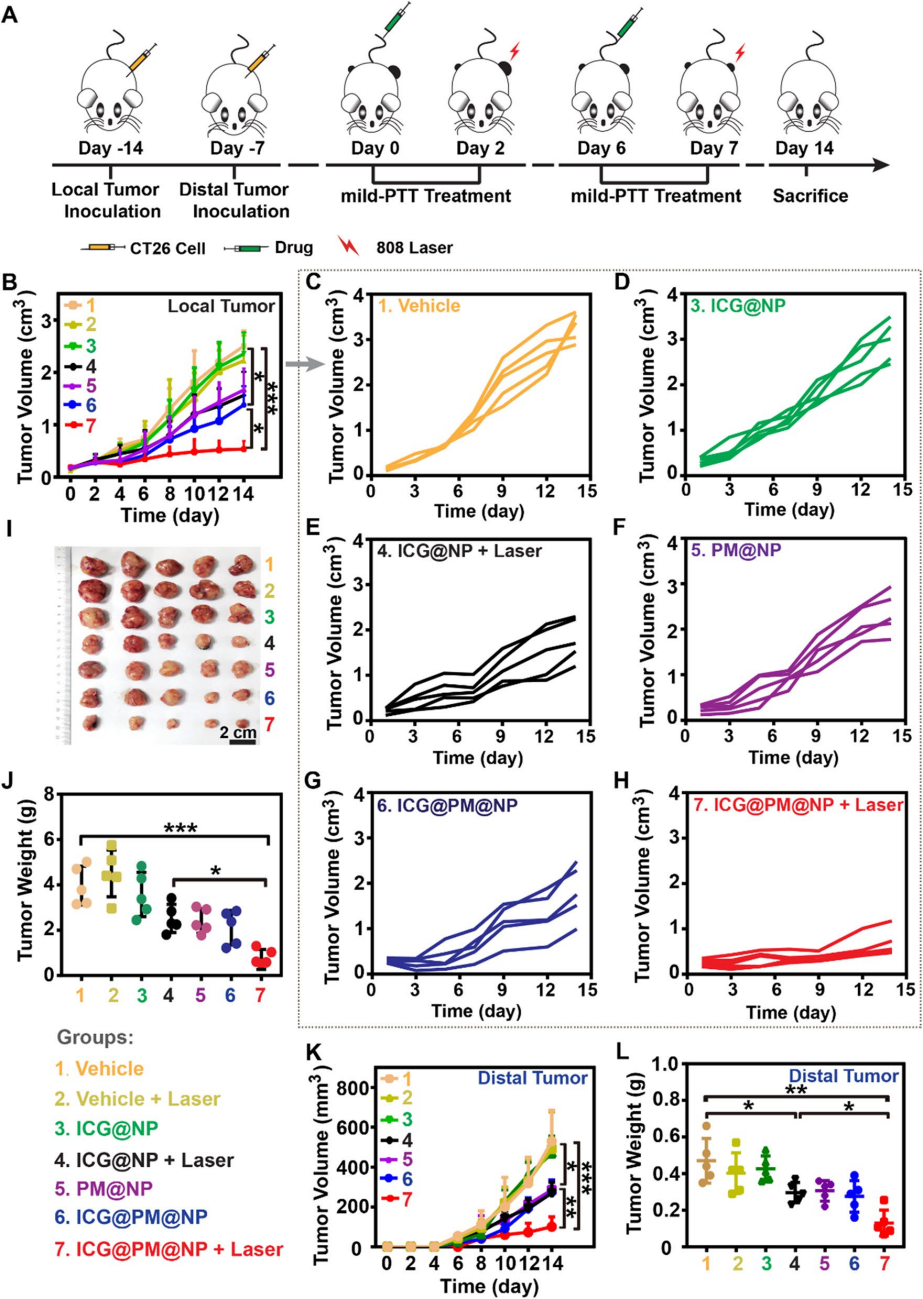
In vivo antitumor efficacy of ICG@PM@NP mediated mild-PTT in CT26 tumors. **A** Schematic diagram of CT26 tumor experimental design. **B**–**H** Tumor growth curves of CT26 tumor-bearing mice after the following treatments (n = 5): 1. Vehicle; 2. Vehicle + Laser; 3. ICG@NP; 4. ICG@NP + Laser; 5. PM@NP; 6. ICG@PM@NP; 7. ICG@PM@NP + Laser. **I** Photo of collected CT26 tumors after various treatments, scale bar = 2 cm. **J** Weight of collected CT26 tumors on Day 14 (n = 5). **K** Distal tumor growth curves of CT26 tumor-bearing mice (n = 5). **L** Weights of excised distal CT26 tumors (n = 5). Statistical analysis was performed via the two-tail Student’s *t*-test. * *p* < 0.05; ** *p* < 0.01; *** *p* < 0.001

To evaluate the antitumor efficacy of ICG@PM@NP mediated mild-PTT, the CT26 tumor model was established (Fig. 7A). As Fig. 7B–H showed, the growth of CT26 tumors treated with PM@NP or ICG@PM@NP was slightly slower than that of the vehicle group contributed to the ability of PM in causing tumor cell death. Meanwhile, ICG@PM@NP mediated mild-PTT treatment almost completely inhibited the growth of CT26 tumors, while ICG@NP mediated mild-PTT did not possess such obvious function, indicating that PM can improve the antitumor efficacy of mild-PTT (Fig. 7). On Day 14 after treatment, the mice were sacrificed to weight CT26 tumors. As shown in Fig. 7I, J, the average tumor weight of PBS-treated mice was about 7 times heavier than the mild-PTT mediated by ICG@PM@NP (4.17 ± 0.96 g *vs* 0.64 ± 0.47 g). In contrast, for ICG@NP mediated mild-PTT, it was only about 1.6 times heavier (4.17 ± 0.96 g *vs* 2.48 ± 0.64 g) (Fig. 7I, J). Therefore, mild-PTT mediated by ICG@PM@NP had the most obvious tumor growth inhibitory effect.

Encouraged by the extraordinary ability of ICG@PM@NP mediated mild-PTT to enhance the antitumor immune response, we next investigated the inhibition of distal CT26 tumor growth by the established active immune response. As shown in Fig. 7K, after ICG@PM@NP mediated mild-PTT treatment, a significantly slower tumor growth rate was observed compared with the vehicle group, whereas ICG@NP mediated mild-PTT did not cause such obvious effect (Fig. 7K). In addition, the growth of the distal tumors in ICG@PM@NP and PM@NP treated mice was slightly slower than that in the vehicle group, which may be due to the antitumor effect of PM loaded in these nanoparticles (Fig. 7K). On Day 14, the mice were sacrificed to collect tumors, which showed similar results as the distal tumor growth indicated (Fig. 7L). More importantly, no significant mice body weight changes were observed after ICG@PM@NP administration (Additional file 1: Fig. S11), indicating that ICG@PM@NP had good biocompatibility in vivo. Thus, ICG@PM@NP mediated mild-PTT also possessed an obvious ability in inhibiting the growth of distal tumors due to the immunogenic environment induced by ICG@PM@NP mediated-PTT.

### Inhibition of local tumor growth and tumor metastasis by ICG@PM@NP mediated mild-PTT in 4T1 tumors

**Fig. 8:**
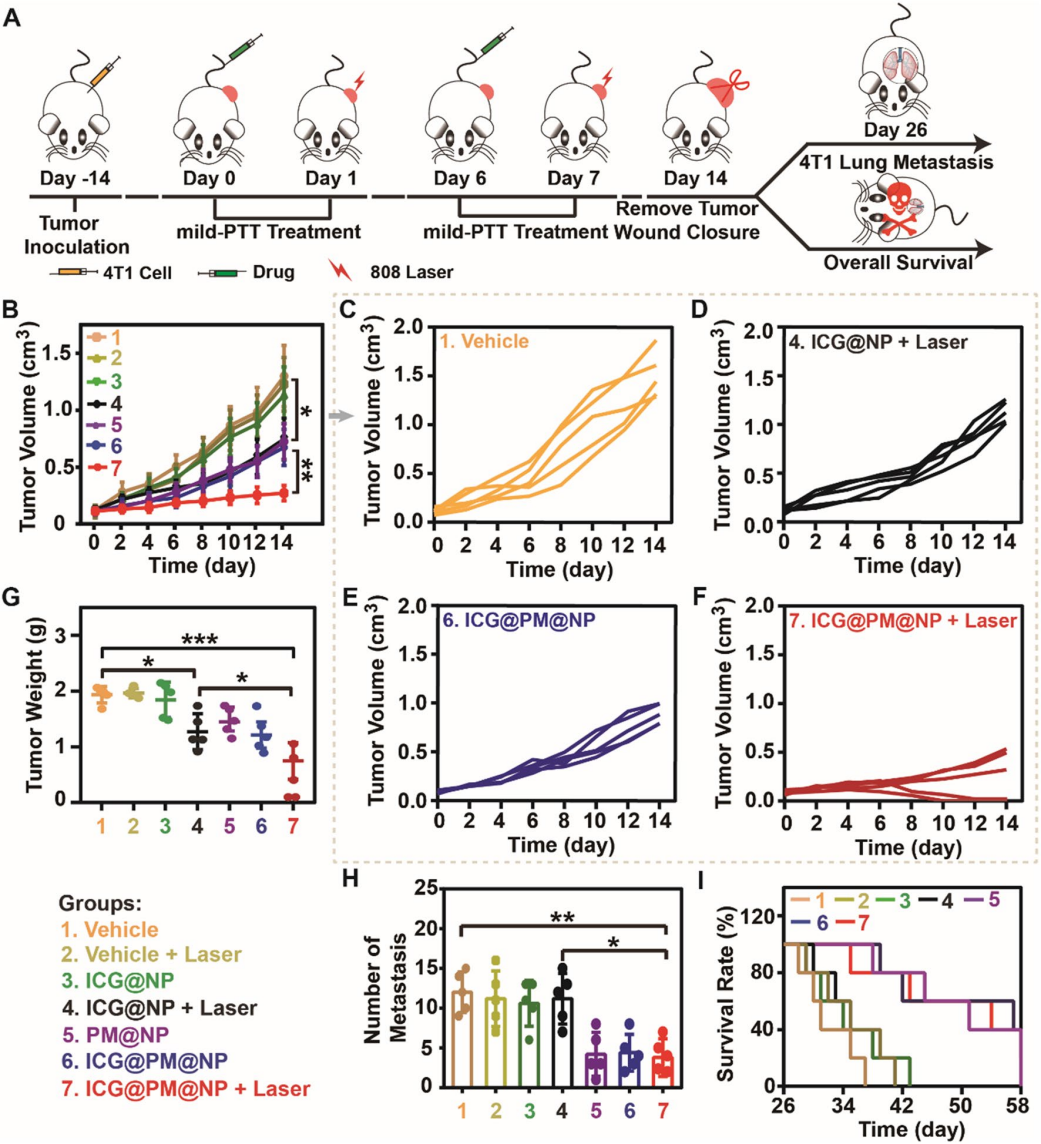
Inhibition of local tumor growth and tumor metastasis by ICG@PM@NP mediated mild-PTT in 4T1 tumors. **A** Schematic diagram of 4T1 tumor experimental design. **B**–**F** Tumor growth curves of 4T1 tumor-bearing mice after the following treatments (n = 5): 1. Vehicle; 2. Vehicle + Laser; 3. ICG@NP; 4. ICG@NP + Laser; 5. PM@NP; 6. ICG@PM@NP; 7. ICG@PM@NP + Laser. **G** Weight of collected 4T1 tumors on Day 14 (n = 5). **H** The number of 4T1 metastatic foci in the lungs of Balb/C mice on Day 26 (n = 5). **I** The survival rate of 4T1 tumor-bearing mice (n = 5). Statistical analysis was performed via the two-tail Student’s *t*-test. * *p* < 0.05; ** *p* < 0.01; *** *p* < 0.001

In addition to CT26 tumors, the general effectiveness of ICG@PM@NP mediated mild-PTT in inhibiting local tumor growth was also studied in 4T1 tumors (Fig. 8). Consistent with the results of CT26 tumors mentioned above, the local 4T1 tumor growth of ICG@PM@NP mediated mild-PTT was most significantly inhibited compared with the vehicle group, while ICG@NP mediated mild-PTT treatment group had no such significant tumor growth inhibition effect (Fig. 8B–F; Additional file 1: Fig. S12). In addition, the weight of 4T1 tumors in the ICG@PM@NP mediated mild-PTT treatment group on Day 14 was only about 30% of the tumor weight in the vehicle group (Fig. 8G; Additional file 1: Fig. S13). Moreover, no significant changes in the body weight were observed after ICG@PM@NP administration (Additional file 1: Fig. S14), consistent with that of CT26 tumor treatment. Therefore, ICG@PM@NP mediated mild-PTT can generally inhibit the local tumor growth of different tumors, including CT26 tumors and 4T1 tumors.

As mentioned above, ICG@PM@NP can effectively inhibit tumor metastasis in vitro by inducing mitochondrial function to induce the upregulation of pAMPK protein (Fig. 4). It was speculated that ICG@PM@NP may also have the ability to reduce tumor metastasis. To demonstrate this, the 4T1 lung metastasis model was established and treated with various formulations (Fig. 8A). As shown in Fig. 8H and Additional file 1: Fig. S15, when treated with PM@NP or ICG@PM@NP, the number of 4T1 lung metastases was significantly reduced, but ICG@NP did not cause this effect. According to the wrapping of PM in the ICG@PM@NP, mild-PTT mediated by ICG@PM@NP efficiently inhibited tumor metastasis as PM@NP did. Moreover, the overall survival time of mice was significantly prolonged by treating with mild-PTT mediated by ICG@PM@NP instead of mild-PTT mediated by ICG@NP (35.0 ± 5.6 Days *vs* 52.4 ± 13.5 Days) (Fig. 8I; Additional file 1: Fig. S16). Taken together, mild-PTT mediated by ICG@PM@NP could effectively inhibit tumor metastasis and make up for the shortcomings of traditional mild-PTT in this respect.

### In vivo toxicity tests of ICG@PM@NP

**Fig. 9:**
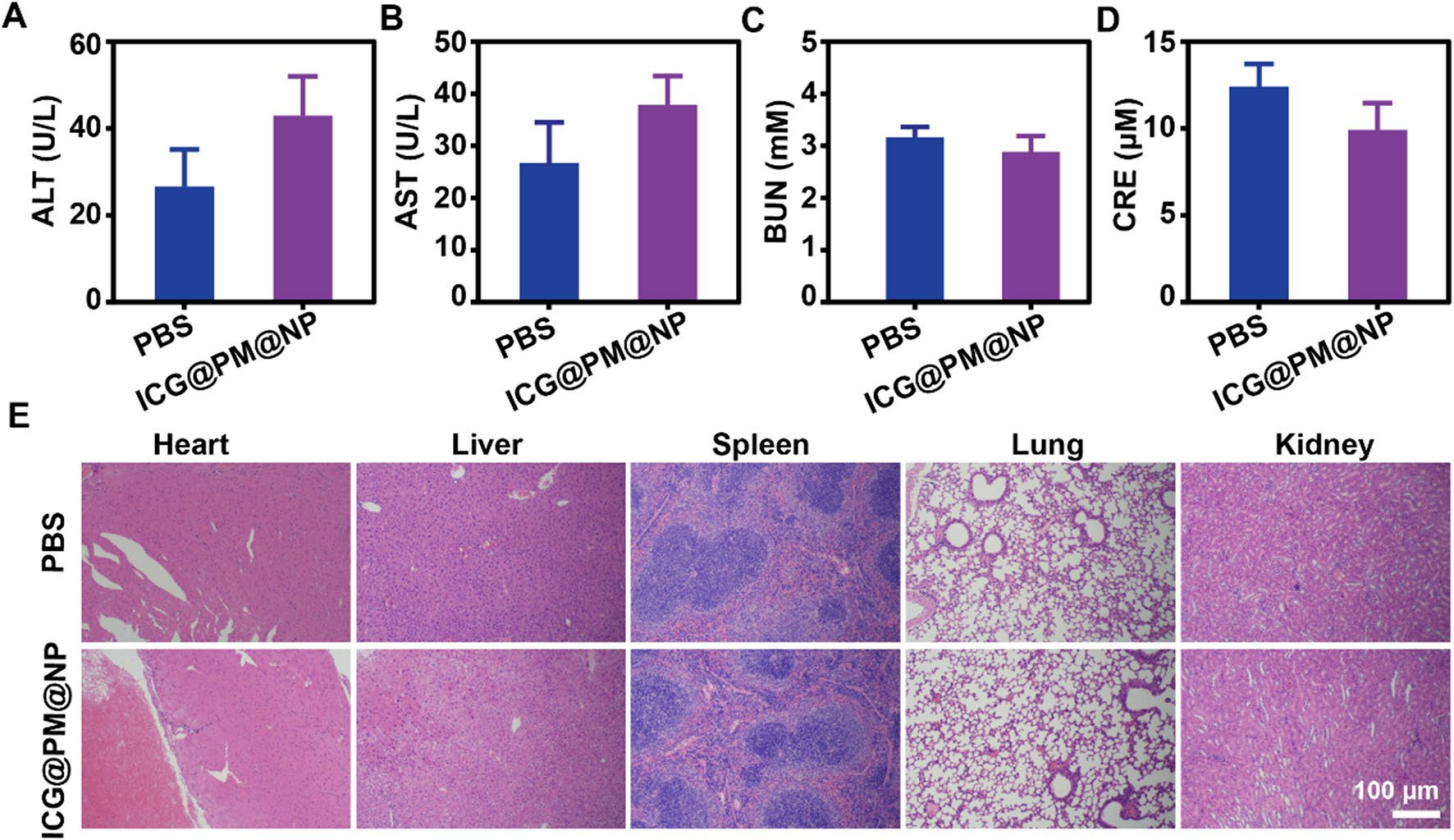
Toxicity tests of ICG@PM@NP on Balb/C mice. **A**, **B** Serum biochemistry data of ALT and AST reflecting the liver function (n = 3). **C**, **D** Serum biochemistry data of BUN and CRE reflecting the kidney function (n = 3). **E** H&E staining images of Liver, Heart, Spleen, Kidney, and Lung on Day 14, scale bar = 100 μm. Data were demonstrated as mean ± SD. Statistical analysis was performed via the two-tail Student’s *t*-test

The safety of ICG@PM@NP was studied by monitoring the renal function, liver function, and H&E staining of the main organs of Balb/C mice (Fig. 9). The liver function after ICG@PM@NP treatment detected by aspartate aminotransferase (AST) and alanine aminotransferase (ALT) was not influenced (Fig. 9A, B). The detection of kidney function markers (blood urea nitrogen, BUN, and creatinine, Cre) also showed that ICG@PM@NP also did not induce any toxicity to the renal function (Fig. 9C, D). Besides, compared with PBS-treated mice, H&E staining also indicated that ICG@PM@NP caused no side effects to normal tissues, including heart, liver, spleen, lungs, and kidneys, showing that ICG@PM@NP possessed no long-term significant toxicity in normal tissues (Fig. 9E). Moreover, results also showed that ICG@PM@NP possessed no hemolytic effect (Additional file 1: Fig. S17). All these results above indicated that ICG@PM@NP with ideal biological safety may could solve the toxicity problem of free PM.

## Discussion

PM is mainly utilized as an oral hypoglycaemic agent for antidiabetic therapy, which has not been designed to reprogram tumor immune microenvironment before. In this study, the molecular mechanism of PM pro-mediated AMPK phosphorylation and subsequence down-regulated PD-L1 was confirmed using AMPK signaling pathway inhibitor Compound C (Fig. 1), showing its high specificity. However, the serious toxicity of PM like acidosis obviously limited its wide usage. To selective deliver PM in tumors to avoid the unbearable toxic and side effect, ICG@PM@NP, a tumor microenvironment responsive nanoplatform, was prepared by the two-step bio-mineralization method for the safe delivery of PM (Scheme 1) [42]. Consistently, ICG@PM@NP activated the PD-L1 regulating function of PM in response to H_2_O_2_ high expression tumor microenvironment, realizing the targeted phosphorylation of AMPK through tumor mitochondrial function inhibition (Fig. 2). Thereby, ICG@PM@NP enabled targeted synergistic effect of mild-PTT triggered by ICG and selected down-regulation of PD-L1 triggered by PM, leading to the localized immune-metabolism intervention (Fig. 3). The immunogenic cell death induced by mild-PTT could then be simultaneously amplified via down-regulation of PD-L1 protein, resulting in increased CD3^+^, CD4^+^ and CD8^+^ T cell infiltration in tumors to reverse immunosuppression microenvironment (Fig. 6). Meanwhile, the expression of two downstream tumor invasion-related signaling proteins of the AMPK pathway (TGF-β and vimentin), was effectively decreased by PM released from ICG@PM@NP, resulting in lowered tumor metastasis risk and prolonged mice survival time that could not be realized by conventional mild-PTT alone (Figs. 4 and 8).

Currently, mild- PTT has demonstrated its remarkable capacity of conquering these obstacles and has shown excellent performance in bacterial elimination, wound healing, and cancer treatments [30]. To further enhance its efficacy, a lot of strategies were discovered, among which the heat shock proteins (HSPs) inhibition were the most widely used. Under hyperthermia conditions of 5 °C higher than the body temperature, tumor cells unusually overexpress HSP70 and HSP90 to improve the heat tolerance and stability for protecting themselves [30, 46]. Therefore, regulating the expression of intracellular HSP70 or HSP90 like ATP production inhibition through mitochondrial function disruption as Pei Wang et al. [46] did can achieve improved mild-temperature PTT efficacy. Considering the fact that ATP production after PM treatment could be obviously reduced, PM or ICG@PM@NP may also process the ability to decrease the HSP70 and HSP90 expression in tumors. Thus, apart from effective PD-L1 regulation, HSP70 or HSP90 may also work partly to enhance the efficacy of mild-PTT mediated by ICG@PM@NP.

NF-κB expression and IFN-γ secretion triggered by most standard clinical treatments, including radiotherapy, chemotherapy, photodynamic therapy, and oncolytic virus therapy [47–52], would generate an immunosuppression microenvironment via limiting T cell activity [53, 54]. Meanwhile, intracellular PD-L1 is also a potential therapeutic target to enhance the efficacy of radiotherapy and chemotherapy in cancers through the inhibition of DNA damage response and repair [55]. Mechanistically different from conventional PD-L1 regulation through PD-L1/PD-1 antibodies or genetic approaches, ICG@PM@NP simulates tumor-specific immunological response pathways through direct inducing mitochondria dysfunction in a persistent manner (Figs. 3 and 6), as the activated AMPK phosphorylation can effectively inhibit PD-L1 expression. It had been validated that ICG@PM@NP had the higher PD-L1 depletion (ca. 4 times) efficiencies in IFN-γ-stimulated CT26 and 4T1 cells relative to those for ICG@NP (Fig. 3J–M). Notably, this high PD-L1 depletion efficiency also enhanced the ICD effect and T cell activity when combined with mild-PTT in mice (Fig. 6E–H). Therefore, ICG@PM@NP enhanced tumor immunogenicity when synergistically with mild-PTT, exhibiting the strongest antitumor T cell immune response, leading to the most effective inhibition of tumor growth and complete prevention of metastasis relative to other controls. Considering the high tumor accumulation and ideal biosafety of ICG@PM@NP, this well-designed H_2_O_2_ responsive nanoplatform is of great potential in the sensitization of mild-PTT for combating primary, distal and metastatic tumors (Figs. 7, 8 and 9).

## Conclusions

In summary, tumor microenvironment responsive nanoplatform ICG@PM@NP that synergizes mild photothermal therapeutic function with selectively tumor-specific immunological response simulator therapy was prepared by two-step bio-mineralization method for efficient photo-immunotherapy of cancer. ICG and PM were effectively loaded into Alb@MnO_2_, thereby enhancing the accumulation of ICG and PM in tumors. The selectively released PM down-regulated the expression of PD-L1 protein by effectively inhibiting tumor mitochondria function, resulting in a significant increase of CD3^+^, CD4^+^, and CD8^+^ infiltration in the tumors. Meanwhile, ICG@PM@NP effectively reduced the expression of two downstream proteins related to tumor invasion (TGF-β and vimentin) in the AMPK protein pathway, thereby reducing the risk of tumor metastasis and prolonging the survival time of mice. These two aspects mediated by PM loaded in ICG@PM@NP made up for the shortcomings of mild-PTT in inhibiting tumor metastasis and inducing immunosuppressive microenvironment, which represents a unique type of immunotherapeutic nanocomplexes that selectively reprogramming the tumor immunosuppressive microenvironment via targeted mitochondria dysfunction. Such design for stimulating tumor-specific immunological response can be generalized for mitochondria dysfunction of many other immuno-regulating drugs (such as PapaVerine, Methylene Blue, Chlormethine Hydrochloride, and Chloroquine) by delivering with other therapeutic agents through the tumor microenvironment responsive nanoplatform. Accordingly, our study not only provides a new combinational therapeutic modality but also opens new opportunities to advance the immunoregulation method in cancer therapy.

## Materials and methods

### Materials

All the chemical reagents were purchased from Aladdin Biochemical Technology Co., Ltd. (Shanghai, China) and used without further purification or modification unless otherwise specified. Fetal bovine serum (FBS), RPMI 1640 medium, DMEM medium, and trypsin–EDTA were purchased from Gibco-Brl (Grand Island, NY, USA). Rabbit anti-mouse or anti-human PD-L1 antibody, E-cadherin antibody, Vimentin antibody, goat anti-rabbit IgG HRP and β-actin antibody were purchased from Affinity Biosciences Inc (USA), AMPK antibody, pAMPK antibody, and TGF-β antibody were purchased from Cell Signaling Technology (USA). All biochemical reagents were used without further purification.

### Cells and animals

Murine colon cancer cells CT26, murine breast cancer cells 4T1, human lung adenocarcinoma cells A549, and human breast cancer cells MCF-7 were obtained from the American Type Culture Collection (ATCC). All these cells were cultured in 1640 medium containing 10% fetal bovine serum (FBS) and 1% penicillin/streptomycin at 37 °C under 5% CO_2_ in a cell incubator (Thermal Fisher Inc, USA).

Balb/c female mice (6–8 weeks old, 20 g) were purchased from the animal experimental center of Wenzhou Medical University (Zhejiang, China). All procedures strictly complied with the ethical and legal requirements under the Administration Committee of Experimental Animals in Zhejiang Province and were approved by the Ethics Committee of Wenzhou Medical University.

### PD-L1 expression test for PM treating cells

To evaluate the effects of PM in down-regulating PD-L1 expression in vitro, 1 × 10^6^ CT26 cells, 1 × 10^6^ 4T1 cells, or 1 × 10^6^ MCF-7 cells were seeded in a 6 cm cell culture dish. After incubation for 24 h, the cells were incubated with fresh medium containing different concentrations of PM for 24 h or incubated with 30 μM PM for another 2 h, 10 h, or 24 h. Afterward, the protein in these cells was extracted and the expression of β-actin, PD-L1, AMPK, and pAMPK protein was measured by western blot assay. All the standardized protein expression levels in the tumor tissues or cells were quantified by ImageJ.

To evaluate the mechanism of PM-mediated PD-L1 lower expression in vitro, 1 × 10^6^ CT26 cells were seeded in a 6 cm cell culture dish. After incubation for 24 h, the cells were incubated for another 24 h with fresh medium containing the following drugs: 60 μM PM; 10 μM Compound C; 60 μM PM + 10 μM Compound C. Then, the protein of cells was extracted and the expression of β-actin, PD-L1, AMPK, and pAMPK protein was measured by western blot assay. All the standardized protein expression levels in the tumor tissues or cells were quantified by ImageJ.

### Synthesis and characterization of ICG@PM@NP

ICG@PM@NP was obtained via a two-step bio-mineralization method according to the previously reported methods [24, 40]. Briefly, 760 mg albumin was dissolved in 20 mL deionized water. Then, 3 mg/mL of ICG was added into the Alb solution at 37 ℃ under vigorous stirring to form the ICG@Alb complex for 2 h. After that, 1 mL manganese chlorides solution (MnCl_2_·4H_2_O, 12 mg/mL) was added into the ICG@Alb suspension. Simultaneously, the pH value of the mixture was adjusted to 10.0 by 1.0 M NaOH to obtain the ICG@Alb@MnO_2_ intermediate. Then, PM (400 μL; 10 mg/mL) was added to the above solution dropwise. After another 5 min, 300 μL KMnO_4_ (12 mg/mL) solution was injected into the solution of ICG@Alb@MnO_2_ intermediate. The solution of KMnO_4_ and PM were alternately added dropwise into the solutions for three cycles to form the final ICG@PM@NP. All the free reagents were removed by an ultrafiltration tube (Millipore 8400, ultrafiltration membrane MW: 30 kDa). PM@NP and ICG@NP nanoparticles were prepared with a similar method as shown in Additional file 1: Fig. S1.

The morphology of ICG@PM@NP was observed by transmission electron microscopy (TEM, Thermo Fisher Scientific, USA). The size distribution and zeta potential of ICG@PM@NP, PM@NP, and ICG@NP were detected by Zetasizer Nano ZS ZEN3600 (Malvern, UK). The loading efficiency and encapsulation efficiency of ICG and PM in these nanoparticles were measured by UV–VIS spectrometer (Lambda 25, PerkinElmer, USA) and high-performance liquid chromatography-mass spectrometry (HPLC–MS) (Agilent 1290 Infinity II and 6135 LC–MS, Agilent, USA), respectively.

To characterize drug release behavior of ICG and PM from ICG@PM@NP, 2 mL ICG@PM@NP was added into the dialysis bag (30 kDa) and submerged into 100 mL different PBS buffers (pH 7.4, pH 7.4 plus 100 μM H_2_O_2_). At the pre-determined time points, the dialysate of each group was obtained. Then, the released ICG and PM concentration was detected by a UV–vis spectrophotometer (Lambda 25, PerkinElmer, USA) and HPLC–MS, respectively.

To characterize drug release behavior of ICG from ICG@PM@NP in PBS at different pH situations, 2 mL ICG@PM@NP was added into the dialysis bag (30 kDa) and submerged into 100 mL different PBS buffers (pH 7.4, pH 6.5 or pH 5.5). At the pre-determined time points, the dialysate of each group was obtained. Then, the released ICG concentration was detected by a UV–VIS spectrophotometer (Lambda 25, PerkinElmer, USA).

### The photo-thermal efficacy of ICG@PM@NP in vitro

To investigate the photo-thermal heating capacity of ICG@PM@NP, 25, 50, 100 μg/mL free ICG or ICG@PM@NP (calculated by ICG concentration) were placed in 2 mL tube. Then, these tubes were irradiated with a 1 W/cm^2^ 808 nm laser for 60 s. The infrared thermal imaging and the temperature were measured by an infrared thermal camera (FLIR E50, USA). PBS was used as control in the same conditions.

To investigate the heating capacity of ICG@PM@NP, 100 μg/mL ICG@PM@NP (calculated by ICG concentration) was placed in a 2 mL tube. Then, these tubes were irradiated with a 1 W/cm^2^ 808 nm laser for 30 s and cooled for 5.5 min. The infrared thermal imaging and the temperature were measured by an infrared thermal camera (FLIR E50, USA). Finally, the ICG@PM@NP was irradiated for five times.

To investigate the photothermal heating capacity of different nanoparticles, ICG@NP, PM@NP, and ICG@PM@NP, ICG@PM@NP (ICG and PM concentration: 25 μM ICG, 50 μM PM) were placed in 2 mL tube. Then, these tubes were irradiated with a 1 W/cm^2^ 808 nm laser for different time. The temperatures of these tubes were measured by an infrared thermal camera (FLIR E50, USA).

### Cell uptake of ICG@PM@NP

CT26 cells were seeded into 24 well plates with a density of 5 × 10^4^ per well. After incubation for 24 h, the cell medium was changed with fresh medium containing ICG@NP, PM@NP, or ICG@PM@NP (ICG concentration: 20 μM) and incubated for another 2 h or 4 h. Then, the cells were washed three times with PBS to remove free drugs, followed by 4% paraformaldehyde fixing for 15 min and DAPI staining for 5 min. Finally, the cells were observed by confocal laser scanning microscopy (CLSM, Nikon, Japan).

### Cell cytotoxicity assay

Cell cytotoxicity test was conducted by using Cell Counting Kit-8 assay. Generally, CT26 cells were seeded into the 96 well plates with 5 × 10^3^ cells per well and cultured overnight. Then, the cells were pre-treated with different concentrations of ICG@NP, PM@NP, and ICG@PM@NP for 12 h. Afterward, the cells were irradiated for 60 s with 808 nm laser (1 W/cm^2^) twice with 10 min intervals. The pre-treated cells without laser irradiation were used as control. After 24 h, the cell viability was detected by the CCK-8 assay (Beyotime Biotechnology Co., Ltd, Shanghai, China).

### Calreticulin (CRT) expression on the tumor cell membrane

CT26 cells were seeded in 6 well plates with a density of 5 × 10^4^ per well. After incubation for 12 h, the cells were pre-treated with fresh medium containing PBS, ICG@NP, PM@NP, and ICG@PM@NP (ICG concentration: 40 μM; PM concentration: 80 μM) for 6 h. Afterward, the cells were irradiated for 60 s with 808 nm laser (1 W/cm^2^) twice with 10 min intervals. After incubation for another 4 h, the cells were stained with anti-CRT antibody (dilution factor 1:1000; Affinity Biosciences Inc., USA) for 4 h, followed by DAPI staining for another 5 min and the fluorescence signals were observed by confocal laser scanning microscopy (CLSM, Nikon, Japan).

### Transwell migration assay

Boyden chamber transwell migration assays were performed according to the manufacturer’s protocol (Corning Incorporated, USA). Briefly, 4T1 cells or A549 cells were firstly pretreated by the following treatments (ICG concentration: 15 μM; PM concentration: 30 μM): 1. Vehicle; 2. ICG@NP; 3. ICG@NP + Laser; 4. PM@NP; 5. ICG@PM@NP; 6. ICG@PM@NP + Laser. Laser groups were irradiated for 60 s with 808 nm laser (1 W/cm^2^) twice with 10 min intervals. After 6 h, 2 × 10^5^ of the treated cells filled with serum-free culture medium were seeded into transwell. The bottom wells were filled with cell culture medium containing 15% fetal bovine serum (FBS) and 1% penicillin/streptomycin. After 24 h, the cells were fixed with 4% formalin and then stained by 5% crystal violet in 70% ethanol. Finally, migrated cells were imaged and counted.

### PD-L1 expression test for ICG@PM@NP treating cells

Briefly, 1 × 10^6^ CT26 cells, 1 × 10^6^ 4T1 cells, or MCF-7 cells were seeded into the 6 cm cell plate. After incubation for 24 h, the cells were incubated with fresh medium containing the following formulations for 24 h: PM@NP, ICG@NP, and ICG@PM@NP (ICG concentration: 20 μM; PM concentration: 40 μM). Afterward, the proteins in these cells were extracted and the expression of PD-L1 protein was detected by western blot assay.

To evaluate induced PD-L1 overexpression in vitro after mild PTT, 1 × 10^6^ CT26 cells were seeded in the 6 cm cell plate. After incubation for 24 h, the cells were incubated with fresh medium containing the following drugs: ICG@NP and ICG@PM@NP (ICG concentration: 20 μM; PM concentration: 40 μM). 4 h later, laser groups were irradiated for 60 s with 808 nm laser (1 W/cm^2^) for twice with 10 min interval. After another 20 h, the proteins in these cells were extracted and the expression of PD-L1 protein was detected by western blot assay.

### Bio-distribution of ICG@PM@NP in vivo

CT26 tumors were established in the left axillary region of Balb/C mice by subcutaneous inoculation with CT26 cells as the local tumor (0.5 × 10^6^ cells per mouse). When the tumor size reached 100 mm^3^, CT26 tumor-bearing mice (n = 3) were intravenously injected with ICG@NP or ICG@PM@NP (ICG concentration: 2.5 mg/kg). The NIR fluorescence images of ICG were acquired by a multi-mode optical live imaging system (IVIS Lumina XRMS Series III, PerkinElmer, USA) at determined time points. Then, at 24 h and 48 h, the mice were sacrificed to collect the tumors and major tissues (hearts, livers, spleens, lungs, and kidneys). The ICG and PM were quantified by the ultraviolet near-infrared detection (Lambda 25, PerkinElmer, USA) and high-performance liquid chromatography-mass spectrometry (HPLC–MS) (Agilent 1290 Infinity II and 6135 LC–MS, Agilent, USA), respectively.

### The photothermal efficacy of ICG@PM@NP in vivo

CT26 tumors were established in the left axillary region of Balb/C mice by subcutaneous inoculation with CT26 cells as the local tumor (0.5 × 10^6^ cells per mouse). When the tumor size reached 100 mm^3^, CT26 tumor-bearing mice (n = 3) were intravenously injected with PBS, free ICG, or ICG@PM@NP (ICG concentration: 5 mg/kg). After 24 h, the mice were irradiated with 808 nm laser (1 W/cm^2^) for 60 s twice with 10 min interval. The infrared thermal imaging of the mice and the temperature of these tumors at the endpoint were measured by an infrared thermal camera (FLIR E50, USA).

### PD-L1 expression test for ICG@PM@NP treating CT26 tumors

CT26 tumors were established in the left axillary region of Balb/C mice by subcutaneous inoculation with CT26 cells as the local tumor (0.5 × 10^6^ cells per mouse). When the tumor size reached 100 mm^3^, CT26 tumor-bearing mice (n = 3) were intravenously injected with ICG@NP or ICG@PM@NP (ICG concentration: 5 mg/kg; PM concentration: 10 mg/kg). 24 h later, the laser groups were irradiated 60 s with 808 nm laser (1 W/cm^2^) twice with 10 min intervals. After another 24 h, the proteins in these cells were extracted and the expression of PD-L1 protein was detected by western blot assay.

### In vivo antitumor efficacy of ICG@PM@NP in CT26 tumors

CT26 subcutaneously transplanted tumor models were used to evaluate the antitumor efficacy of ICG@PM@NP mediated mild-PTT. Firstly, tumors were established in the left axillary region of Balb/C mice by subcutaneous inoculation with CT26 cells as the local tumor (0.5 × 10^6^ cells per mouse). On Day 7, CT26 cells were inoculated at the right axillary region of the Balb/C mice as distal tumors (1 × 10^6^ cells per mouse). When the local tumor size reached 100 mm^3^, PBS, ICG@NP, PM@NP, and ICG@PM@NP were given severally through tail vein injection (ICG concentration: 5 mg/kg; PM concentration: 10 mg/kg). These drugs were given in the corresponding groups every 7 days twice. 24 h later, the laser groups were irradiated 60 s with 808 nm laser (1 W/cm^2^) twice with 10 min intervals. The body weight and tumor volume (primary tumor and distal tumor) of each mouse were monitored every 2 days for 2 weeks. The tumor volumes were calculated according to the following formula: A*B^2^*0.5, where A is the major axis and B is the minor axis of the tumors. On Day 14, all the mice were sacrificed and tumor tissues were collected, weighed, and photographed.

### In vivo antitumor metastasis efficacy of ICG@PM@NP in 4T1 tumors

4T1 subcutaneously transplanted tumor models were used to evaluate the antitumor efficacy of ICG@PM@NP mediated mild-PTT. Firstly, tumors were established in the left axillary region of Balb/C female mice by subcutaneous inoculation with 4T1 cells (0.5 × 10^6^ cells per mouse). When tumor size reached 100 mm^3^, PBS, ICG@NP, PM@NP, and ICG@PM@NP were severally injected into the mice through the tail vein (ICG concentration: 5 mg/kg; PM concentration: 10 mg/kg). These drugs were given in the corresponding groups every 7 days twice. 24 h later, the laser groups were irradiated 60 s with 808 nm laser (1 W/cm^2^) twice with 10 min intervals. The body weight and tumor volume of each mouse were recorded every 2 days for two weeks. On Day 14, all of the tumors were surgically removed and the wound stitched up for the following study. On Day 26, part of the mice (5 of 10) were sacrificed to collect the lungs to observe and analyze the lung metastasis foci of 4T1 tumors. The other part of the mice (5 of 10) were observed to analyze the overall survival time.

### Evaluation of tumor apoptosis and proliferation in vivo

CT26 subcutaneously transplanted tumor models were used to evaluate the anti-tumor efficacy of ICG@PM@NP mediated mild-PTT. Firstly, tumors were established in the left axillary region of Balb/C mice by subcutaneous inoculation with CT26 cells as the local tumor (0.5 × 10^6^ cells per mouse). When tumor size reached 100 mm^3^, PBS, ICG@NP, and ICG@PM@NP were severally injected into the mice through the tail vein (ICG concentration: 5 mg/kg; PM concentration: 10 mg/kg). 24 h later, the laser groups were irradiated 60 s with 808 nm laser (1 W/cm^2^) twice with 10 min intervals. To analyze the T cells infiltration and distribution in the primary tumors, mice were sacrificed after various treatments to collect tumors for evaluating the infiltration of T cells. Briefly, the tumor tissues embedded in OCT were cut into 6 μm slices and fixed with cold acetone. Then, the tumor slices were stained by FITC anti-mouse CD3, FITC anti-mouse CD4, and FITC anti-mouse CD8 antibodies (dilution factor 1:500, Biolegend Inc, USA) overnight at 4 ℃. After staining with DAPI for another 5 min, fluorescence images of tumor slides were collected by CLSM.

Besides, hematoxylin and eosin (H&E) staining were also performed for histopathological analysis. In addition, a one-step Tunel assay kit (Beyotime, Shanghai, China) was also used to stain tumor tissue slices and tumor apoptosis was analyzed by CLSM (Nikon, Japan).

For Ki67-based cell proliferation ability assessment, the samples were fixed and incubated overnight with rabbit anti-mouse Ki67 antibody (dilution factor 1:2000, Affinity Biosciences Inc, USA) and then incubated with Alexa Fluor 594 labeled goat anti-rabbit IgG (H + L) (Thermo Scientific, USA) for 1 h at room temperature. Finally, the fluorescence images were observed by a CLSM.

### Biosafety evaluation of ICG@PM@NP

To evaluate the biocompatibility of ICG@PM@NP, ICG@PM@NP (ICG concentration: 5 mg/kg; PM concentration: 10 mg/kg) were intravenously injected into healthy mice every seven days for a total of two injections. On Day 14, mice were sacrificed to collect the blood and major organs. To evaluate the hepatorenal toxicity of ICG@PM@NP, the serum levels of aspartate aminotransferase (AST), alanine aminotransferase (ALT), serum creatinine (CRE), and urea nitrogen (BUN) were detected by assay kits purchased from the Jiancheng Bioengineering Institute (Nanjing, China). The major organs were collected, fixed with 4% neutral formalin, and embedded in paraffin, followed by H&E staining for histological analysis.

### Statistical analysis

Statistical analysis was performed via the one-way ANOVA test. Meanwhile, post hoc analysis was performed using the Wilcoxon rank-sum test with a Bonferroni correction when needed. * *p* < 0.05 was considered statistically significant; ** *p* < 0.01 and *** *p* < 0.001 were extremely significant; NSD, no significant difference.

## Supplementary Information

**Additional file 1.** Additional figures S1–S17.

## Data Availability

All data used to generate these results is available within the paper and the Supporting Information.
