# Supplementary material for: Selectively down-regulated PD-L1 by albumin-phenformin nanoparticles mediated mitochondrial dysfunction to stimulate tumor-specific immunological response for enhanced mild-temperature photothermal efficacy
Source: J Nanobiotechnology. 2021 Nov 18;19:375. doi: 10.1186/s12951-021-01124-8 (PMC8600872; doi:10.1186/s12951-021-01124-8)
Supplement: Supplementary file 1 — Additional file 1. Additional figures S1–S17. [file 12951_2021_1124_MOESM1_ESM.docx]

**Selectively Down-regulated PD-L1 by Albumin-Phenformin Nanoparticles Mediated Mitochondrial Dysfunction to Stimulate Tumor-specific Immunological Response for Enhanced Mild-Temperature Photothermal Efficacy**

Zaigang Zhou ^a, #^, Ning Jiang ^d, #^, Jiashe Chen ^a, #^, Chunjuan Zheng ^a^, Yuanyuan Guo

^b^, Ruirong Ye ^d,^*, Ruogu Qi ^b,^*, Jianliang Shen ^a, c, e,^*

^a^ State Key Laboratory of Ophthalmology, Optometry and Vision Science, School of Ophthalmology and Optometry, School of Biomedical Engineering, Wenzhou Medical University, Wenzhou 325027, China.

^b^ Department of Biochemistry and Molecular Biology, School of Medicine & Holistic Integrative Medicine, Nanjing University of Chinese Medicine, Nanjing, 210023, China.

^c^ Wenzhou Institute, University of Chinese Academy of Sciences, Wenzhou 325001, China.

^d^ Faculty of Life Science and Technology, Kunming University of Science and Technology, Kunming 650500, China

^e^ Oujiang Laboratory, Wenzhou, Zhejiang, 325000, China

^#^ These authors contributed equally to this paper.

* Corresponding authors:

[shenjl@wiucas.ac.cn](mailto:shenjl@wiucas.ac.cn) (Jianliang Shen);

[rqi@njucm.edu.cn (Ruogu](mailto:rqi@njucm.edu.cn%20(Ruogu) Qi) ;

yerr@mail2.sysu.edu.cn (Ruirong Ye).

**
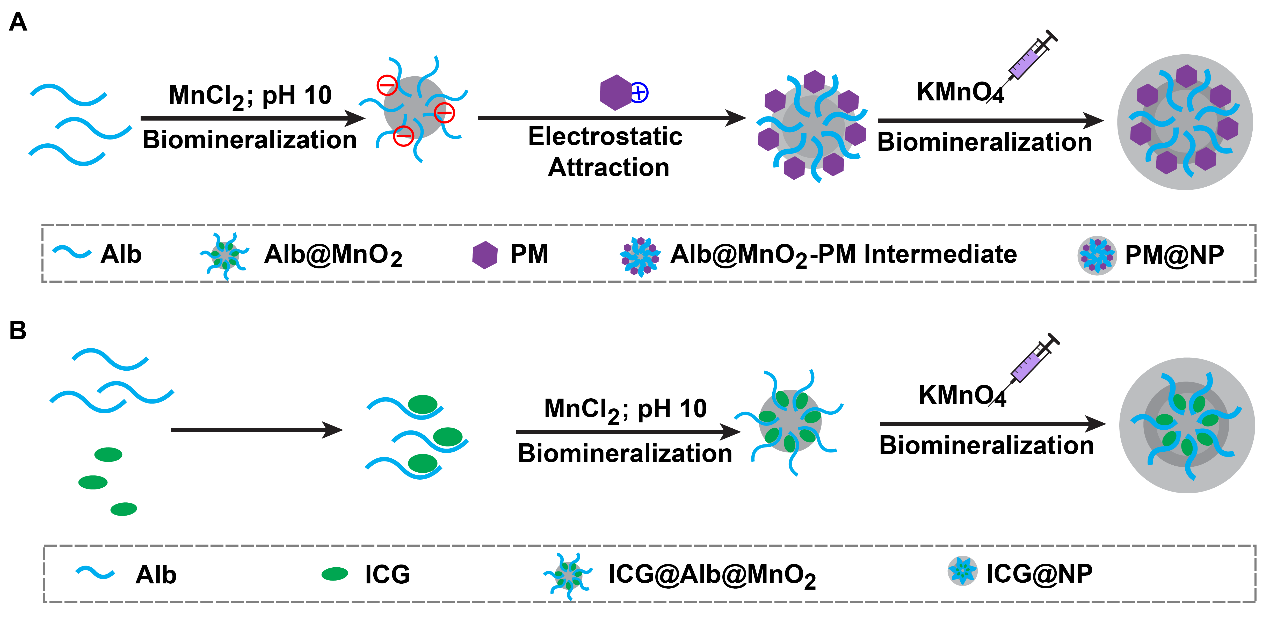
Figure S1.** (A) The synthesis route of PM@NP. (B) The synthesis route of ICG@NP.

**
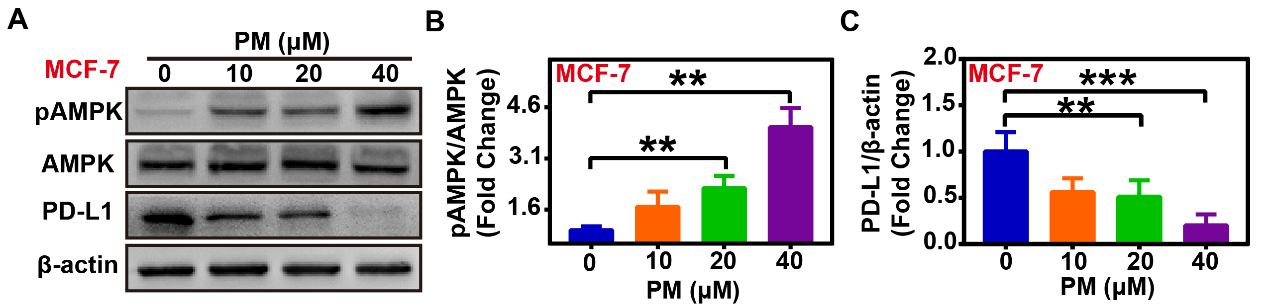
**

**Figure S2.** Effects of PM on PD-L1 expression in MCF-7 human tumor cells *in vitro*. (A) Effects of different concentrations of PM on PD-L1, AMPK, and pAMPK protein expression in MCF-7 cells *in vitro* detected by western blot after treatments for 24 h (n = 3). (B-C) Quantification of PD-L1 protein or pAMPK protein expression levels in western blot by Image J in MCF-7 cells (n = 3). Data were demonstrated as mean ± SD. Statistical analysis was performed via the two-tail Student’s *t*-test. ** *p* < 0.01; *** *p* < 0.001.

**
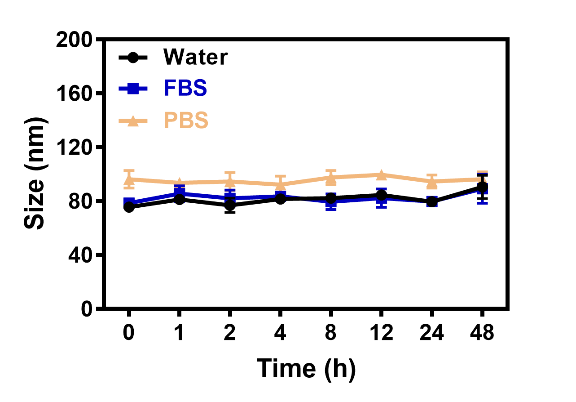
**

**Figure S3.** The stability of ICG@PM@NP in water, 10% FBS, and PBS detected by DLS (n = 3).


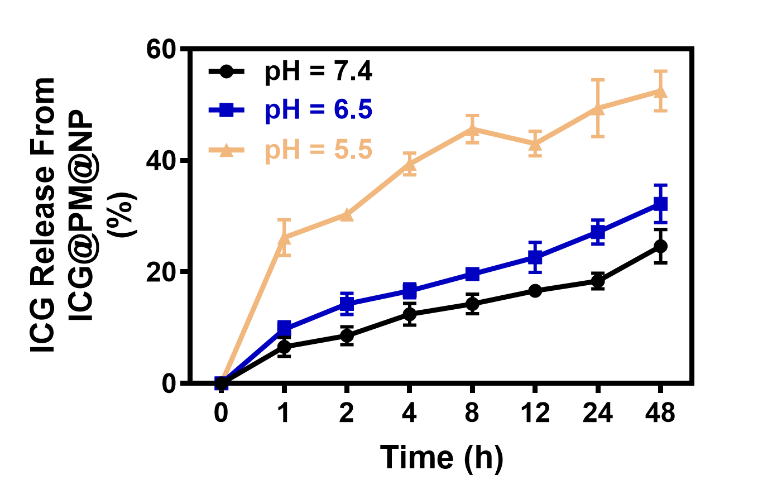


**Figure S4.** Release of ICG from ICG@PM@NP in PBS at different pH (n = 3).


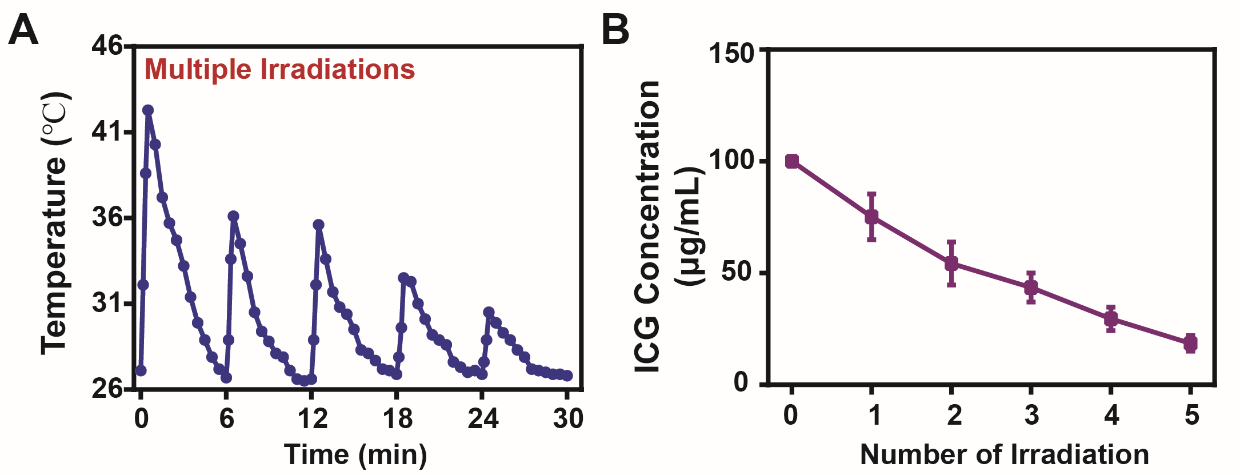


**Figure S5.** (A) The heating curve of ICG@PM@NP (100 μg/mL, calculated by ICG content) after multiple irradiations. (B) Detection of the remaining ICG concentration after multiple irradiations (n = 3).


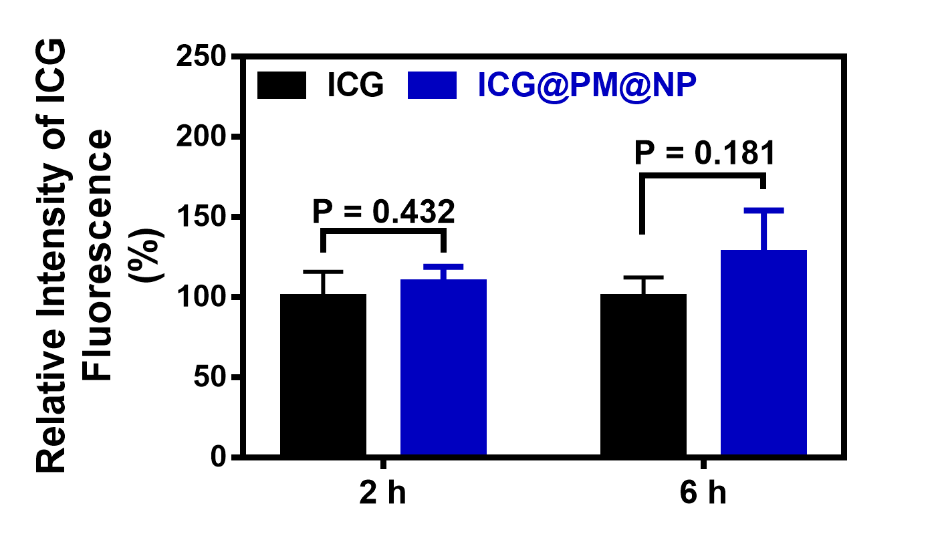


**Figure S6.** Detection of the relative ICG fluorescence intensity at different time after free ICG or ICG@PM@NP treatment (n = 3). Data were demonstrated as mean ± SD. Statistical analysis was performed via the two-tail Student’s *t-*test.


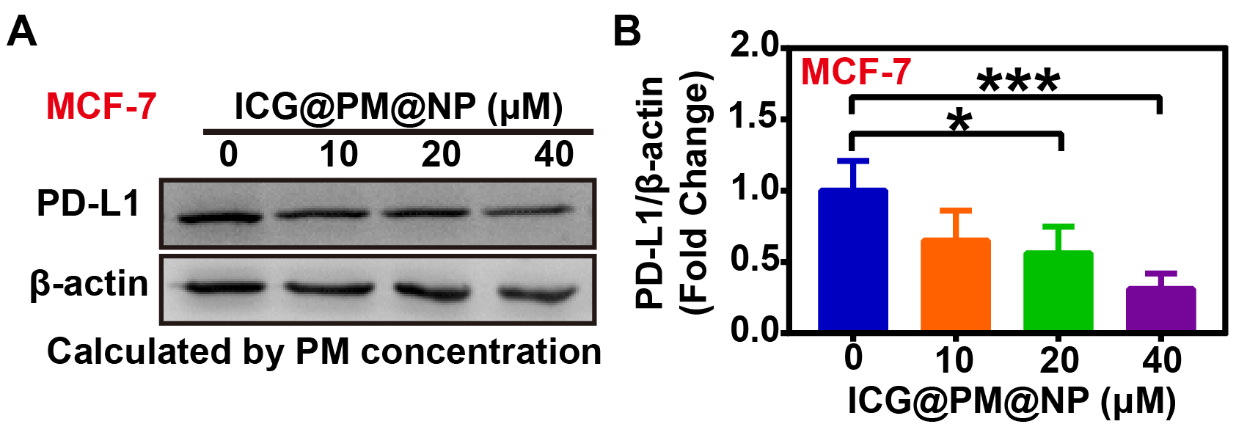


**Figure S7.** Effects of ICG@PM@NP on PD-L1 expression in MCF-7 human tumor cells *in vitro*. (A) Effects of different concentrations of ICG@PM@NP on PD-L1 protein expression in MCF-7 cells *in vitro* detected by western blot after treatments for 24 h (n = 3). (B-C) Quantification of PD-L1 protein expression levels in MCF-7 cells by Image J (n = 3). Data were demonstrated as mean ± SD. Statistical analysis was performed via the two-tail Student’s *t*-test. * *p* < 0.05; *** *p* < 0.001.

**
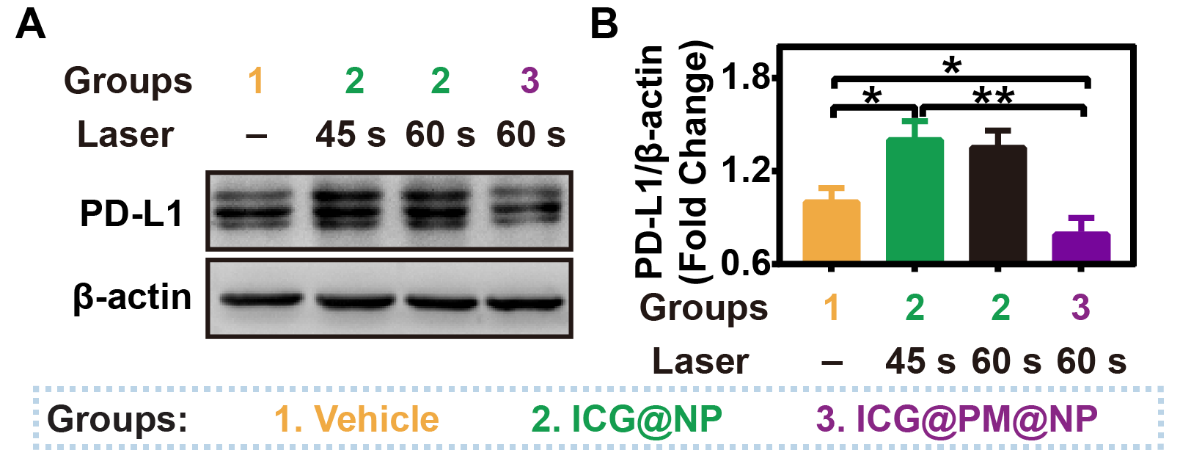
**

**Figure S8.** Effects of ICG@PM@NP mediated mild-PTT on the PD-L1 expression in CT26 tumor cells *in vitro*. (A) Effects of ICG@PM@NP on the mild-PTT induced PD-L1 protein over-expression in CT26 cells *in vitro* detected by western blot after treatments for 24 h (n = 3). (B) Quantification of PD-L1 protein expression levels in western blot by ImageJ in CT26 cells (n = 3). Data were demonstrated as mean ± SD. Statistical analysis was performed via the two-tail Student’s *t*-test. * *p* < 0.05; ** *p* < 0.01.

**
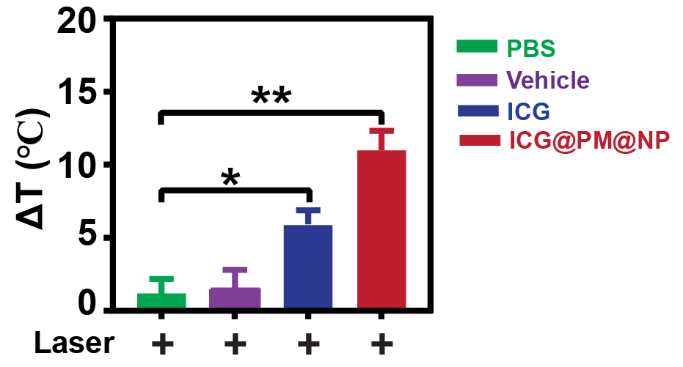
**

**Figure S9.** Increased temperature of the CT26 tumors irradiated 60 seconds at 1 W/cm^2^ at 24 h after different treatments. Data were demonstrated as mean ± SD. Statistical analysis was performed via the two-tail Student’s *t*-test. * *p* < 0.05; ** *p* < 0.01.


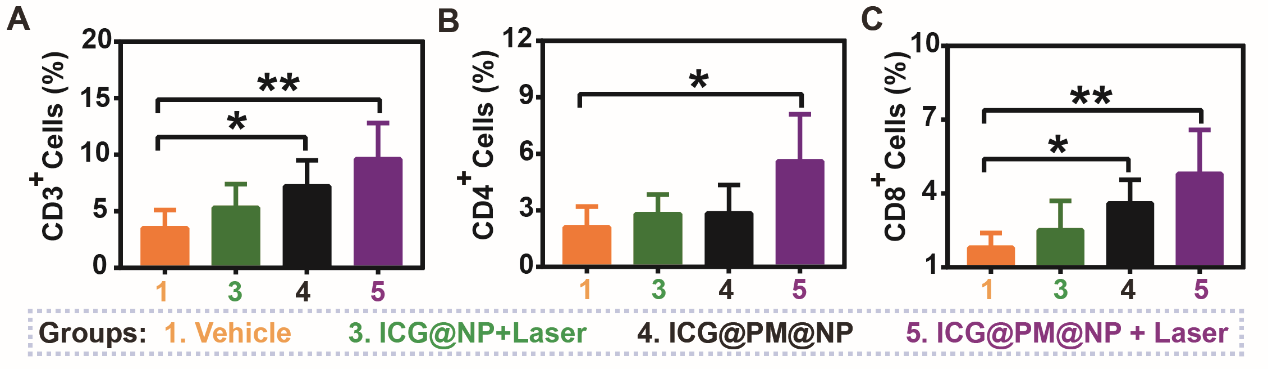


**Figure S10.** Quantification of CD3^+^ T cells, CD8^+^ T cells, and CD4^+^ T cells in CT26 tumor slices to evaluate the effects of ICG@PM@NP mediated mild-PTT on T cell infiltration (n = 3). Data were demonstrated as mean ± SD. Statistical analysis was performed via the two-tail Student’s *t-*test. * *p* < 0.05; ** *p* < 0.01.


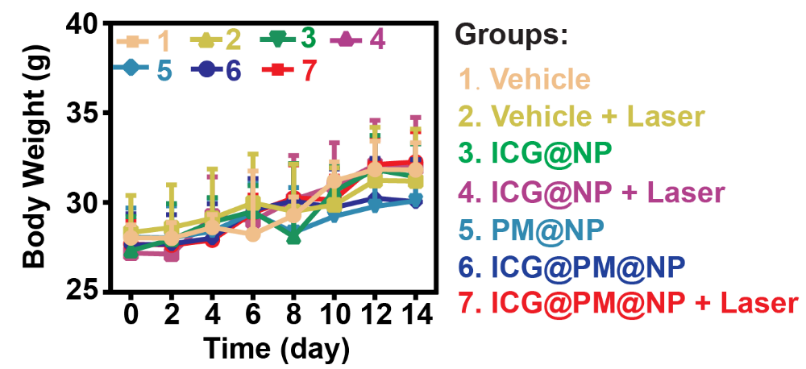


**Figure S11.** Body weight curves of CT26 tumor-bearing Balb/C post-treatments (n = 5).


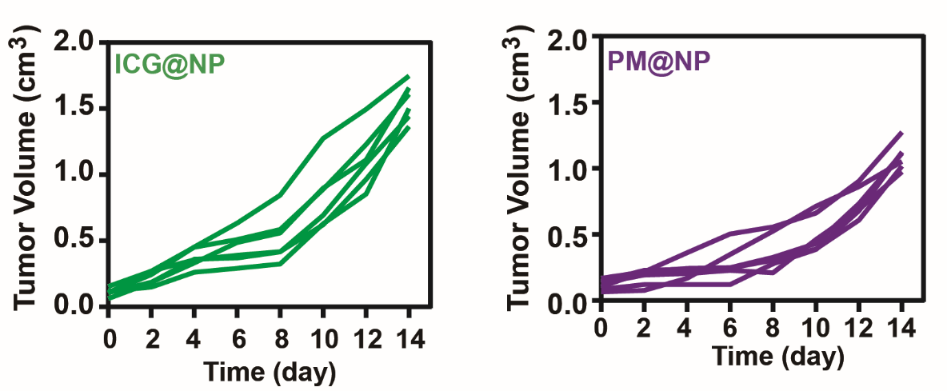


**Figure S12.** Tumor growth curves of 4T1 tumor-bearing mice after treatments (n = 5).


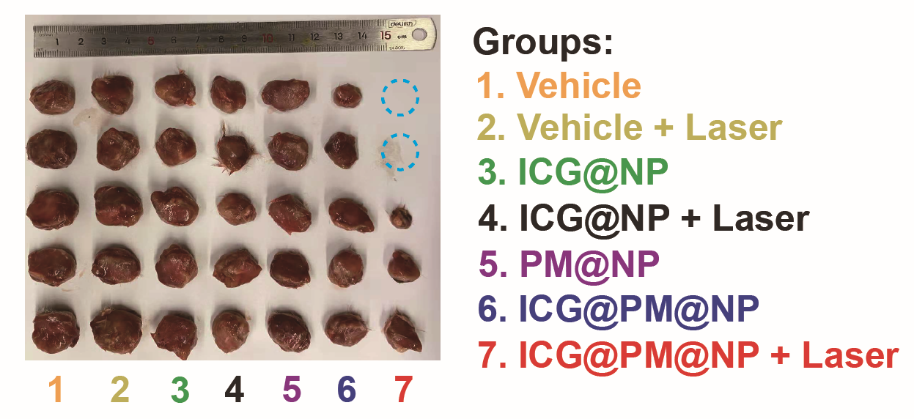


**Figure S13.** Photo of collected 4T1 tumors after the following treatments (n = 5): 1. Vehicle; 2. Vehicle + Laser; 3. ICG@NP; 4. ICG@NP + Laser; 5. PM@NP; 6. ICG@PM@NP; 7. ICG@PM@NP + Laser.


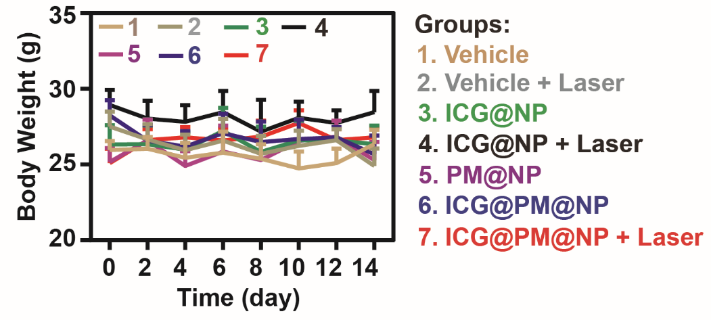


**Figure S14.** Body weight curves of 4T1 tumor-bearing Balb/C post-treatments (n = 5).


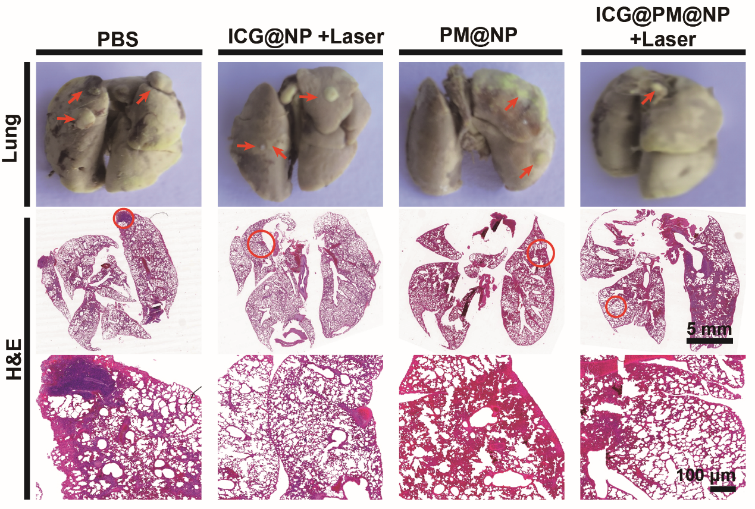


**Figure S15.** Photo and H&E staining of collected lungs with 4T1 metastasis foci after various treatments (n = 5).


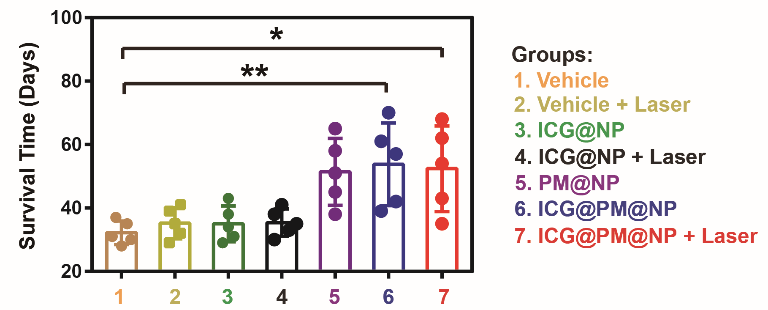


**Figure S16.** Survival time of 4T1-bearing mice after treatments (n = 5). Data were demonstrated as mean ± SD. Statistical analysis was performed via the two-tail Student’s *t*-test. * *p* < 0.05, ** *p* < 0.01.


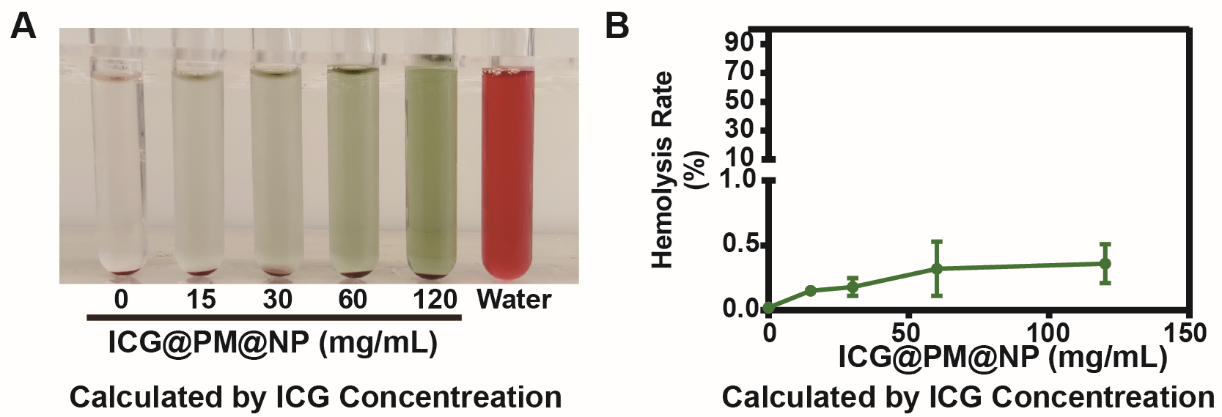


**Figure S17.** (A) Representative image of the hemolytic effect after treatment of different concentrations of ICG@PM@NP (calculated by ICG content). (B) Hemolytic rate induced by ICG@PM@NP at different concentrations (n = 3).
